# Development of Scaffolds from Bio-Based Natural Materials for Tissue Regeneration Applications: A Review

**DOI:** 10.3390/gels9020100

**Published:** 2023-01-23

**Authors:** Murugiah Krishani, Wong Yen Shin, Hazwani Suhaimi, Nonni Soraya Sambudi

**Affiliations:** 1 Faculty of Integrated Technologies, Universiti Brunei Darussalam, Jalan Tungku Link, Gadong BE1410, Brunei; 2 Department of Chemical Engineering, Universitas Pertamina, Simprug, Jakarta 12220, Indonesia

**Keywords:** tissue engineering, scaffold, fabrication techniques, tissue regeneration

## Abstract

Tissue damage and organ failure are major problems that many people face worldwide. Most of them benefit from treatment related to modern technology’s tissue regeneration process. Tissue engineering is one of the booming fields widely used to replace damaged tissue. Scaffold is a base material in which cells and growth factors are embedded to construct a substitute tissue. Various materials have been used to develop scaffolds. Bio-based natural materials are biocompatible, safe, and do not release toxic compounds during biodegradation. Therefore, it is highly recommendable to fabricate scaffolds using such materials. To date, there have been no singular materials that fulfill all the features of the scaffold. Hence, combining two or more materials is encouraged to obtain the desired characteristics. To design a reliable scaffold by combining different materials, there is a need to choose a good fabrication technique. In this review article, the bio-based natural materials and fine fabrication techniques that are currently used in developing scaffolds for tissue regeneration applications, along with the number of articles published on each material, are briefly discussed. It is envisaged to gain explicit knowledge of developing scaffolds from bio-based natural materials for tissue regeneration applications.

## 1. Introduction

Tissue regeneration is a dynamic process in which the cells and their surrounding matrix interplay. Further, this process is encouraged by designing biomaterials that adapt to the local cellular signals [1]. Transplantation is the conventional method for tissue regeneration, but donor availability, pain, and risks related to graft rejection and infectious disease are some concerns [2]. Tissue engineering is a modern field that promotes tissue replacement and regeneration substitutes. It is a multidisciplinary field in which a biomaterial such as a scaffold, cells, and growth factors are combined to form a new tissue [3,4]. It also helps to overcome the problems faced during autologous and allogeneic tissue repair, such as inadequacy, donor site dejection, and unbidden immune responses [5]. The scaffold acts as a template in which cells and growth factors are implanted to imitate the extracellular matrix to maintain and restore tissue function. High porosity, pore interconnectivity, biocompatibility, biodegradability, and mechanical properties are indispensable properties that must be considered when designing the scaffold [6]. Besides blood cells, most tissue cells reside in a solid matrix known as the extracellular matrix (ECM). The ECM is an anchor for maintaining a proper structure and providing the tissue with mechanical properties and signaling molecules. Hence, the scaffold selected for engineered tissue should mimic the ECM of that specific tissue [7]. Selecting appropriate cells, isolating and expanding targeted cells, and selecting suitable biomaterial for scaffold designing are factors that thrive in tissue engineering [8]. However, a solitary polymer cannot achieve every single property of a scaffold, so the desired property can be attained by mixing it with a variety of polymers [9]. Along with the selection of material, process technique or fabrication method also provide a more significant impact on the features of the resultant scaffold [10]. This paper provides detailed information on bio-based natural materials and the fabrication techniques currently used to develop scaffolds for tissue regeneration applications.

## 2. Tissue Engineering

**Figure 1:**
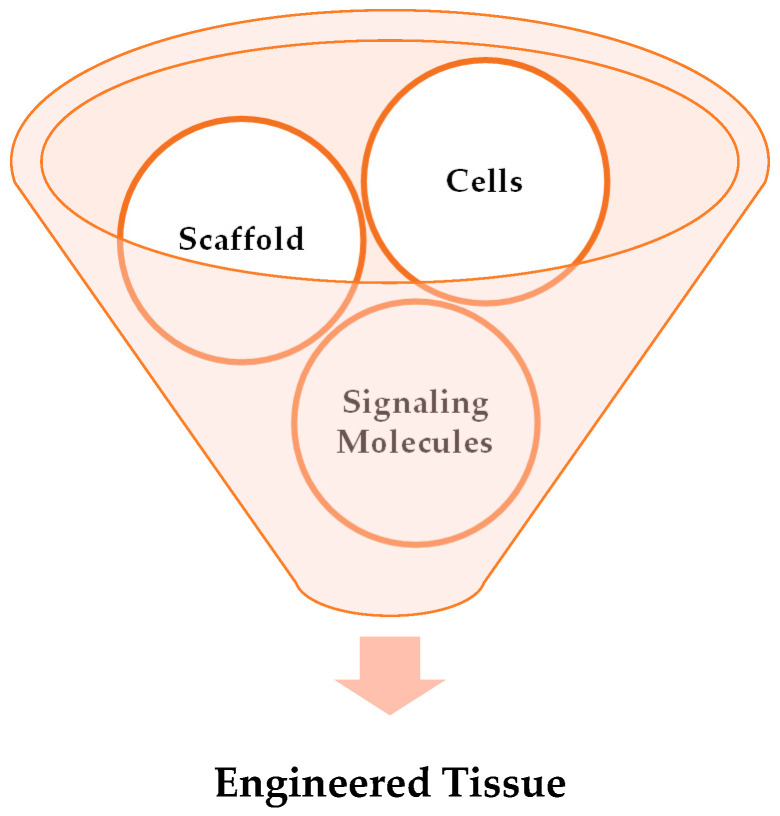
Vital elements of tissue engineering (simplified diagrammatic representation of the basic concept of tissue engineering, i.e., scaffold, cells, and growth-stimulating factors are the three essential parameters responsible in tissue engineering for forming new functional tissue).

Tissue engineering (TE) is a relatively new, unique, multidisciplinary field. It offers new hope to patients by integrating clinical medicine, materials science, cell biology and genetics, and mechanical engineering to design bio-artificial tissues or biological substitutes that restore or regenerate, preserve, and improve damaged tissue or organs [3]. The three essential parameters in tissue engineering, biomaterial scaffolds, cells, and growth-stimulating signals, are known as the “tissue-engineering triad,” as mentioned in Figure 1.

**Figure 2:**
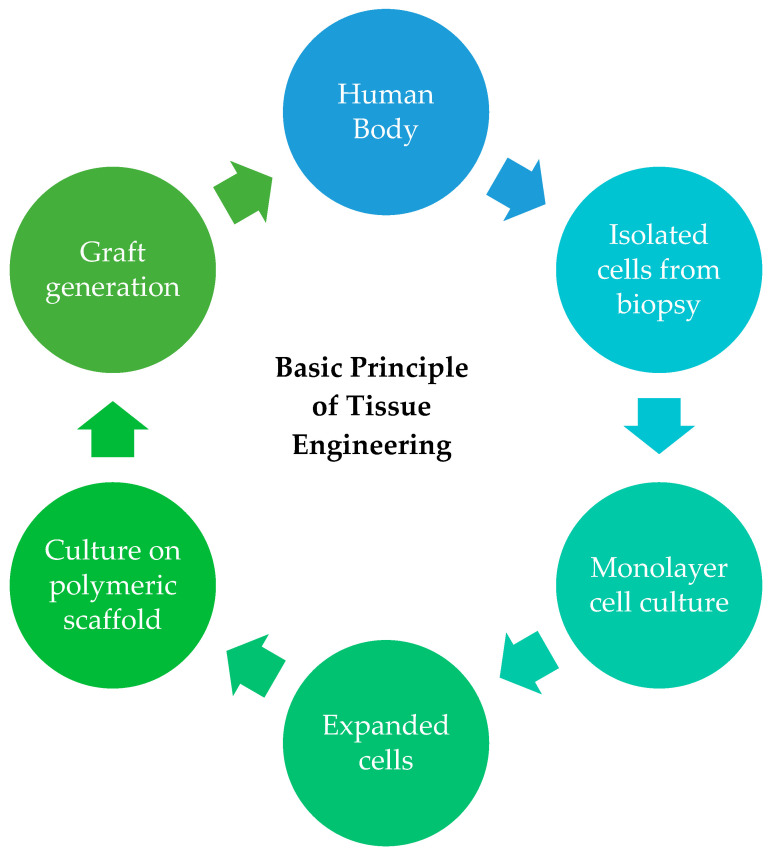
An illustration of the basic principle of TE, which includes cell isolation, cell culture, cell expansion, and tissue grafting into the patient’s body.

The bioreactor uses this triad to imitate a natural environment to reproduce and grow new functional tissues or cellular components. Figure 2 shows an illustration of the basic principle of TE.

Firstly, cells are isolated from a biopsy (allogenic, syngeneic, xenogeneic, or autologous source) and allowed to grow and expand in vitro, in a cell culture system, or in a bioreactor. The expanded cells are then seeded onto a nutrient and growth factors-rich matrix or carrier (scaffold) for structural support. Here, the cells grow, differentiate, and proliferate to form new tissues, then migrate to the carrier to replace the old tissues. Lastly, this TE product will be grafted into the patient to replace the damaged tissues [11].

### 2.1. Key Elements of Tissue Engineering

#### 2.1.1. Cells

The cell is a structural and functional unit of life in all living organisms. Cells performing the same function are grouped to form tissues and create a body system. While designing a TE product, especially for clinical applications, cell source selection becomes a crucial issue, as it determines the success of the tissue generation step. Essentially, the cells isolated for TE applications should fulfill the essential requirement of combining themselves with the selected tissue with different growth factors and cytokines that activate the endogenous tissue regeneration program. However, natural cells have difficulty reproducing the same particular cell type in large quantities. A promising cell source called stem cells is then developed as an alternative. Stem cells can be categorized into embryonic (ESCs), adult (ASCs), and induced pluripotent stem cells (iPSCs). ESCs are pluripotent cells that can differentiate into any desired lineage, but are ethically controversial and have a shortage in teratoma production [12]. ASCs are multipotent cells and are considered more appropriate for TE applications than ESCs. Though ASCs have more limitations in cell differentiation, they are believed to be less prone to rejections after transplantations. Therefore, ASCs are commonly used to isolate tissues such as bone marrow, muscle, adipose tissue, and umbilical cord [13]. iPSCs, on the other hand, are somatic cells in the pluripotent state that exhibit autologous characteristics and fulfill differentiation capacity [12]. Nevertheless, iPSCs are yet to be extensively used due to the need for precise characterizations of reprogramming the somatic cells before clinical applications [14].

#### 2.1.2. Growth Factors

Growth-stimulating signals include growth factors (GFs), which are a heterogeneous group of polypeptides bonded to specific receptors on the cell surface that regulates a heterogeneous group of polypeptides bonded to specific receptors on the cell surface that regulate cellular responses such as cell proliferation and cell survival, as well as the growth of targeted tissues [15]. Some GFs that have been used in TE applications include bone morphogenetic proteins, vascular epithelial growth factor (VEGF), and transforming growth factor-β (TGF-β) [16].

#### 2.1.3. Scaffolds

Scaffolds play an important role in TE applications, serving as a temporary platform or template for providing guidance and structural support to develop new tissues [17]. Scaffolds refer to a three-dimensional (3D) porous biomaterial that provides a favorable environment for cells to repair and regenerate tissues and organs [3]. It serves as a template for tissue defect reconstruction while promoting cell attachment, proliferation, extracellular matrix regeneration, and restoration of nerves, muscles, and bones. In addition, scaffolds can transport bioactive materials such as drugs, inhibitors, and cytokines as a mechanical barrier against the infiltrating native tissues, which may disturb tissue restoration and regeneration [11].

### 2.2. Requirements of Scaffold

#### 2.2.1. Microarchitecture

The microarchitecture of the scaffold includes the porosity and pore size and the interconnectivity between the pores. Firstly, the pore size must be adequate for cell migration and attachment onto scaffolds. This also ensures proper mass transfer of nutrients and waste materials into and out of the cells and tissue or vascularization and infiltration. As suggested by Perić Kačarević et al., a smaller pore size is favorable, between 75 and 100 μm in vitro, while the maximum pore size should lie between 200 and 500 μm in vivo to allow optimal tissue penetration and vascularization [18,19]. Moreover, an interconnecting porous system is required to provide a larger scaffold surface area for cell attachment. In addition, having a higher porosity helps to maximize cell-to-cell interactions, thereby promoting the integration of the engineered tissues with the native tissues [20]. Alonzo et al. suggested a pore network comprising more than 60 percent of pores with pore diameters ranging between 150 and 400 μm and at least 20 percent smaller than 20 μm [21].

#### 2.2.2. Biodegradability

As scaffolds only act as a temporary platform for developing cells or tissues, they should be chemically or enzymatically broken down over time when grafted into living organisms. The rate at which the scaffold materials are broken down is known as biodegradability [3]. Ideally, the biodegradation rate of the scaffold should be proportional to the rate of new bone formation or tissue regeneration. When new tissues are successfully engineered and integrated with host bone, they will replace the biomaterial scaffolds via a “creeping substitution” step [22]. The non-toxic products of the scaffold will then be recycled as metabolites in other biochemical reactions or exit the body without interference with other organs and surrounding tissues [18,20].

#### 2.2.3. Biocompatibility

Furthermore, the scaffold should be highly biocompatible for cell adhesion and proliferation. There should be negligible chronic immune responses to prevent severe inflammatory reactions that might affect healing or cause rejection in the body. Even when inflammatory reactions occur, they should be recovered in no more than two weeks [23,24].

#### 2.2.4. Bioactivity

Scaffold bioactivity refers to its ability to interact with the surrounding cellular components of the engineered tissues. Unlike traditional passive biomaterials, which generally pose low or no interactions with the environment, bioactive scaffolds are designed to enhance proper cell migration or differentiation, tissue regeneration or neoformation, and integration in the host, thereby avoiding processes such as scarring [19]. Moreover, the scaffolds may be attached to cell-adhesive ligands to promote cell attachment, or to physical indicators such as topography to enhance cell morphology and alignment. In addition, bioactive scaffolds may serve as a transporter or reservoir for growth-stimulating signals such as GFs to enhance tissue regeneration [7].

#### 2.2.5. Mechanical Properties

Furthermore, the scaffold materials should pose similar intrinsic mechanical properties as native bones or tissues in the anatomical site of implantation. The mechanical properties of tissue vary in nature, as listed in Table 1. It provides structural support and shape stability and, at the same time, helps to minimize the risk of stress shielding, implant-related osteopenia, and subsequent re-fracture. Moreover, the scaffold should also be strong enough to allow surgical handling during transplantations. Some examples of mechanical properties include elastic modulus, tensile strength, fracture toughness, fatigue, and elongation percentage [7,18,19,25].

**Figure 3:**
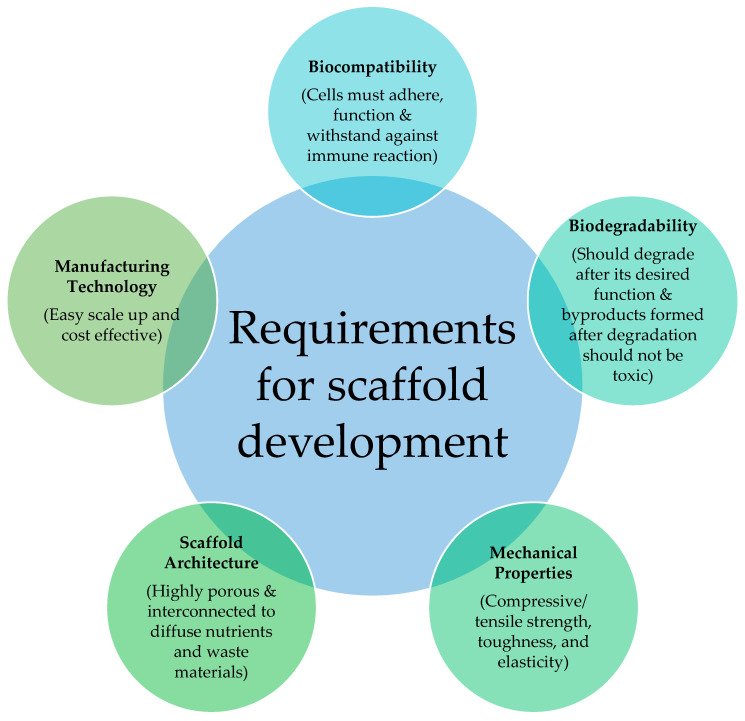
The necessary ideal scaffold requirements include biocompatibility, biodegradability, mechanical properties, scaffold architecture, and manufacturing technology.

Tensile testing and compressive testing are the conventional methods used to characterize the mechanical properties of a scaffold. Compressive/tensile strength, toughness, and Young’s modulus are the important obtained parameters. No limitations for the geometrical structure of the specimen is the biggest advantage of compressive testing over tensile testing. Atomic force microscopy (AFM), dynamic mechanical analysis (DMA), rheometry, and micro indentation are the alternative methods for the characterization of mechanical properties [25]. Elasticity (Young’s modulus), shear strength, and viscoelasticity measurement are some significant mechanical properties in cardiac tissue engineering. Due to its thin geometric structure (µm thickness), it is inadequate for DMA. Hence, viscoelasticity measurement for the cardiac scaffold is incorporated only in a few studies [35]. For the healing process, to endure osteogenic loads, adequate compressive strength is needed in bone tissue engineering [36]. Compared to other tissues, neural tissues have low mechanical stiffness with the range of 0.1KPa for Young’s modulus [37]. Mechanical properties play a crucial role in skin tissue engineering to resist physiological forces such as nerve bundles, vascular networks, and collagen deposition during the wound healing process [38]. Figure 3 depicts the requirements to be considered while developing the scaffold.

#### 2.2.6. Manufacturing Technologies

As stated by Place et al., TE products must be both productive and cost-effective, introducing a potential dichotomy between the need for sophistication and ease of production [39]. While ensuring scaffold efficiency, it is also essential to consider the cost and availability, ensuring scale-up production of the scaffolds is feasible when required. Another key factor to consider is delivering and packaging the scaffolds to the clinicians. Even though clinicians usually prefer off-the-shelf availability to lessen waiting time before implantations, it may not be possible for some tissue types [40]. Therefore, this should be considered while implementing a TE strategy.

### 2.3. Materials Used for Developing Scaffold

The material source for scaffolds should depend on the patient’s status. For instance, patients with cancer or osteoporosis generally experience low bone metabolism; hence, the scaffold material should be non-resorbable. Nevertheless, the material source would come under biomaterials. According to the European Society for Biomaterials (ESB), a biomaterial is a material meant to interface with biological systems to treat, evaluate, augment or replace any tissue, organ or function of the body [18,40]. The four major biomaterials typically used in the fabrication of scaffolds are polymers, bio-ceramics, metals, and carbon-based nanomaterials. As each group has specific advantages and disadvantages, scaffolds may comprise more than one of these biomaterial types [40,41]. Natural polymers, synthetic polymers, bio-ceramics, biodegradable metals, and carbon-based nanomaterials are currently used in scaffold development [1].

#### 2.3.1. Polymer

A polymer is a long-chained macromolecule built up by repeated monomers, and polymer-based biomaterials are considered a good choice for fabricating a scaffold [42]. Polymers are a good candidate in TE applications for their great versatility and flexibility in providing a wide range of mechanical, chemical, and physical properties. They show good biocompatibility, are light in weight, and are resistant to biochemical attack. Moreover, polymers are highly available at a reasonable cost and quickly processed into desired shapes. In addition, the inertness of polymers towards host tissues makes them an eligible candidate for a drug delivery system. Some biomedical applications involving polymers include artificial organs and blood vessels, breast implants, contact lenses, coatings for pharmaceutical tablets and capsules, external and internal ear repairs, cardiac assist devices, and joint replacements [43]. Polymeric biomaterials have been obtained from natural and synthetic polymers, each having pros and cons [44,45]. Carbohydrates such as chitin, cellulose, starch, alginate, and hyaluronic acid and proteins such as collagen, elastin, keratin, gelatin, and fibrin fall under natural polymers, where polyesters such as poly ε-caprolactone (PCL), polylactic acid (PLA), and polyglycolic acid (PGA) and Polyurethanes come under synthetic polymers [44].

1. Natural polymers: Biopolymers are toxic-free, highly biocompatible, easily adhere to cells, and improve proliferation and differentiation. Nevertheless, they have poor mechanical strength and are highly sensitive to elevated temperatures [46]. Biopolymers are also known as natural polymers. Natural polymers are materials that can be obtained from natural sources. They can be categorized into protein-based biomaterials (naturally occurring polymers in the human body such as collagen, fibrin, and elastin) and polysaccharides-based biomaterials (such as silk, chitosan, alginate, and gelatin). They exhibit similar characteristics to soft tissues, showing bioactivity, excellent cell adhesion and growth, and fulfilling biodegradability and biocompatibility. Moreover, they are also known for their wide availability, ecological safety, and modifiability to suit different applications. However, natural sources indicate the requirement of a purification step to avoid foreign immunological responses after implantation. In addition, natural polymers typically show poor physical and mechanical stability, limiting their applications in the load-bearing orthopaedic field [17,43].
2. Synthetic polymers: In contrast to natural polymers, synthetic polymers have good mechanical properties. However, they also have a high risk of immune rejection, and toxic substances such as carbon dioxides are released during degradation, leading to cell damage [40]. Synthetic polymers serve as a more predictable biomaterial providing a wide range of mechanical and physical properties such as degradation rates. If they are synthesized under controlled conditions, they do not pose any immunological risks, and desired characteristics can be brought together. One common synthetic polymer used for BTE applications is aliphatic polyesters, including poly (ε-caprolactone) (PCL) and polylactide (PLA). PCL is a semi-crystalline, biodegradable, and non-toxic polyester that shows hydrophobicity and slow degradation rates of more than 24 months. These problems can be addressed by blending with other polymers or producing composites. In contrast, the porous PLA exhibits high biocompatibility, but shows slow degradation rates of 3–5 years. Thus, PLA is combined with hydroxyapatite (HAp) to improve its mechanical and physical strength [18,43].

#### 2.3.2. Bio-Ceramics

Ceramic materials obtained from natural products, termed bio-ceramics, have been widely used in dental and bone tissue engineering. Bio-ceramics are organic, non-metallic solids with good compatibility, bio-inertness, bioactivity, osteoconductivity, and mechanical strength [18,43]. In addition, bio-ceramics can promote new bone generation and the osteo-potential of scaffolds. However, bio-ceramics are low in elasticity with a brittle surface, limiting their use in implants. Thus, they are usually blended or coated with other materials to improve their elasticity and strength. Among the biomaterials, bio-ceramic scaffolds have been proven to be more successful in treating minor bone defects, such as orthopaedic implants and bone-filling applications. There are three main types of ceramics: bioinert, bioactive, and bioresorbable. Bioinert ceramics include alumina (Al_2_O_3_), Zirconia (ZrO_2_), and pyrolytic carbon; bioactive ceramics include bioglasses (BG) and glass ceramics, while bioresorbable ceramic contains calcium phosphates. Of all three types, the most commonly used ceramics in BTE applications are HAp, tricalcium phosphates (TCP), and their composites [3]. Human bone and teeth are composed of an inorganic compound known as hydroxyapatite, which constitutes calcium, phosphate, and OH radicals with high tensile strength and quickly adheres to host tissues. Many studies revealed that HAp is non-toxic and lacks inflammatory and pyrogenetic response [47].

HAp is a naturally occurring calcium phosphate-based mineral with the chemical formula Ca_10_(PO_4_)_6_(OH)_2_. It is structurally similar to a biological apatite in the human body, known as bone mineral, which makes up approximately 60–70% of human bone tissues on a dry weight basis. It shows similar chemical and physical properties to human bone and dental tissues [48,49]. Hence, some hydroxyapatite-rich natural products such as shells, corals, algae, fish scales, and animal bones are used to develop scaffolds [50,51]. HAp exhibits excellent biocompatibility and bioactivity, high osteoinductivity and osteoconductivity, non-toxicity, and non-inflammatory characteristics. Moreover, it vitalizes growth factors and promotes cell growth and proliferation. HAp is therefore considered a highly potential implant material and bone substitute. Nevertheless, HAp also shows poor mechanical properties, slow resorption and remodeling rates, and slow degradation rates in vivo, making it unsuitable for all BTE applications. Thus, HAp is usually synthesized with other natural or synthetic polymers to create more effective composite scaffolds [19,52].

HAp can be obtained through extractions from natural sources or chemical syntheses, divided into three categories: high-temperature methods, wet methods, and dry methods. Dry methods include mechanochemical methods and solid-state reactions. Here, dry precursors of calcium and phosphate are mixed without any precisely controlled conditions to synthesize HAp. According to Sadat-Shojai et al., this is to ease the mass production of HAp powders. Wet methods include sol-gel, chemical/wet/co-precipitation, hydrothermal, hydrolysis, sonochemical method, and emulsion method. With this method, the morphology and average powder size can be controlled. However, HAp yielded usually exhibits low crystallinity due to low operating temperatures. High-temperature processes include combustion and pyrolysis methods, where samples undergo thermal decompositions. These two methods are rarely used for HAp synthesis due to poor control over the operating conditions [53].

HAp can also be extracted from food wastes or biological sources such as aquatic or marine sources, mammalian bones shell sources, and plant sources [54]. This method is relatively safe, more sustainable, and economical to fabricate HAp, thereby contributing to the economy, environment, and general health. However, it is notable that natural HAp is non-stoichiometric, either calcium or phosphorus-deficient. Generally, calcium positions would be the vacancy, where cations such as Na^+^, Mg^2+^, and Al^3+^ are substituted into the vacant space. Likewise, carbonate ions would replace phosphate or hydroxyl ions, and fluoride ions would substitute in place of hydroxyl ions. These trace elements present in the natural HAp resemble the apatite in human bone, which is crucial in accelerating bone formation and regeneration. For instance, blending 3–5 mol% silicon with synthetic HAp can boost cell growth density, enhancing osteoblast growth. Another example is adding 1–10% of strontium ions in synthetic HAp, which improves osteoblast activity and material differentiation [55]. Calcium carbonate is abundantly found in the exoskeleton of most marine organisms such as corals, sea urchins, and some algae. HAp produced from these exoskeletons are highly porous, have good vascularization and blood supply, and help to form new tissue [56]. Over the past 20 years, extensive research has been done to constantly improve the synthesis methods and introduce new technologies, aiming to develop an ideal HAp composite or scaffold that fulfills all the desired specifications.

#### 2.3.3. Metals

Metals such as stainless steel, cobalt–chromium–molybdenum alloy, aluminium, lead, silver, and titanium alloys have been considered good load-bearing implants because of their excellent quality electrical and thermal conductivity, appropriate mechanical properties, corrosion resistance, biocompatibility, and reasonable cost. However, metals are non-biodegradable. Therefore, researchers introduced the use of biodegradable metals [18]. Biodegradable metals are metals having controlled corrosion properties. They can be grouped into pure biodegradable metals (Mg^−^- and Fe^-^-based), biodegradable alloys, and biodegradable metal matrix composites [43]. Pure biodegradable metal implants have similar mechanical properties to stainless steel and bone and are non-toxic. However, they show slow degradation rates and are incompatible with MRI (Magnetic Resonance Imaging). These problems can be addressed through newly-developed fabrication methods such as casting, electroforming, powder metallurgy, and inkjet 3D printing. Moreover, it is essential to note that the patients implanted with biodegradable metals should not have an iron-related disease, and the patient’s intestines can absorb only Fe^2+^. Thus, any Fe^3+^ released should be first reduced to Fe^2+^ before being absorbed [43].

Biodegradable porous metal scaffolds have attracted researchers in scaffold development by their high compressive strength. Biodegradable metals overcome problems such as innate immune rejection and have good load-bearing capacity during bone healing. However, biodegradable metals such as Mg and their alloys have a high corrosion rate. Recently, scientists have concentrated on the Zn-based alloy system to produce biodegradable metal scaffolds [57,58].

#### 2.3.4. Carbon-Based Nanomaterials

Researchers developed carbon-based nanomaterial scaffolds by combining tissue engineering and nanotechnology to enhance the scaffold’s features. Carbon nanotubes (CNTs), graphene oxide (GO), carbon dots (CDs), fullerenes, and nanodiamonds are some carbon nanomaterials used as scaffolds in tissue engineering. Biocompatibility, mechanical stability, low cytotoxicity, facilitating cell communication, and nutrition delivery are advantages of carbon-based nanomaterials that pull down to use in scaffold development. However, limited biodegradability and potential cytotoxicity are significant drawbacks [59].

**Figure 4:**
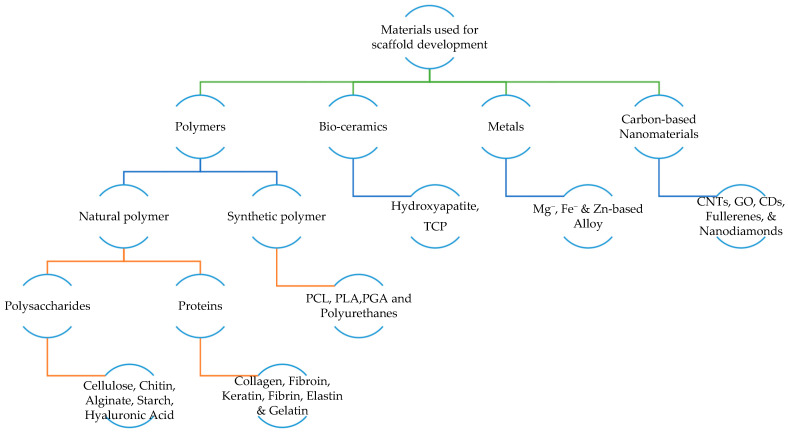
Materials used for scaffold development. (Materials are divided into four broad categories such as polymers, bio-ceramics, metals, and carbon nanomaterials. Classification with a few examples is summarized.)

Carbon-based nanomaterials, including graphene oxide (GO), carbon nanotubes (CNTs), fullerenes, carbon dots (CDs), nanodiamonds (NDs), and their derivatives, are highly potential scaffold materials for bone restoration applications. They are biocompatible, mechanically stable, and commercially available. In addition to that, they show essential qualities such as good biodegradability, efficient cell proliferation and osteogenic differentiations, significant cell growth stimulations, proper mass transfer of nutrients in the scaffold microenvironment, improved cell distributions, and appropriate cell bioactivity. Yet, further studies regarding the low cytotoxicity and the adverse environmental effects of carbon-based nanomaterials are to be conducted before they can be clinically tested and brought into application [1,60]. The materials used for developing the scaffold are summarized in Figure 4.

In recent years, some innovative synthetic scaffolds based on natural products have been developed by applying recombinant DNA technology and advanced genetic engineering. Elastin-like recombinant [ELR] and elastin-like peptides [ELP] are a few scaffolds developed by obtaining the principles of advanced genetic engineering techniques. The Arginine, Glycine, and Aspartic acid (RGD) sequence is an integrin-binding sequence in the ELP scaffolds, which helps in cell adhesion and proliferation. ELR scaffolds constitute a fibronectin domain that helps cell adhesion, particularly in vascular regeneration [61,62]. B. Gurumurthy et al. developed a collagen-based scaffold by incorporating it with an elastin-like polypeptide obtained from genetically modified *Escherichia coli* bacteria and bioglass to examine the osteogenic differentiation [63]. Repeated sequences of elastin and silk blocks are recombinantly combined to form silk-elastin-like protein polymers (SELPs). The hydrogels of SELPs play a vital role in wound healing [62].

On the whole, bio-based polymers have good features such as compatibility, versatility, and adaptability, and are also abundant in nature; they can be obtained from various agricultural resources and biodegradable waste materials. Hence, the processing and synthesis cost is low and environmentally friendly [50,64]. Many researchers have tried to produce scaffolds from natural polymers by keeping this in mind by modifying and enhancing their stability using various fabrication methods [65].

### 2.4. Common Natural Polymers Used in Tissue Regeneration Applications

#### 2.4.1. Cellulose

Cellulose is a fundamental structural unit of the plant cell wall. It is also found in red, green, and brown algae, some fungi, and as an extracellular component in bacteria [66]. Cellulose is a homopolysaccharide composed of D-glucose units connected by β-(1→4) glycosidic bonds [67]. Cellulose is an ideal material for tissue growth. It has several features such as biocompatibility, biodegradability, and cheap cost. It is already used as a scaffolding material in wound repair, cartilage tissue regeneration, differentiating endothelial cells, and bone tissue engineering [60]. Scaffolds developed based on bacterial cellulose are widely used in various biomedical applications [68]. Based on recent research, the performance of the material toward cell growth or biocompatibility is mentioned in Table 2.

#### 2.4.2. Chitin and Chitosan

Chitin is the second most common polysaccharide globally, followed by cellulose. It exists in the exoskeleton of arthropods such as crabs, shrimps, lobsters, insects, prawns, and fungal cell walls. Chitin comprises repeated units of 2-(acetylamino)-2-deoxy-D-glucose. Chitin and chitosan are differentiated by a degree of deacetylation. Chitin has various biomedical applications in tissue engineering due to its outstanding properties such as non-toxicity, biocompatibility, biodegradability, and chelating of metal ions. It also supports cell adhesion, differentiation, and migration. Chitin also has structural similarity with N-glycosaminoglycans, essential components of connective tissues; hence, it is a good option for skin tissue regeneration. Further, it is also used in dental, bone, and cartilage implants [3,114,115]. Mokhtari et al. have developed a scaffold hydrogel by combining chitosan with collagen and aldehyde-modified nanocrystalline cellulose loaded with gold nanoparticles, showing a potential application in tissue engineering [116].

#### 2.4.3. Alginate

**Figure 5:**
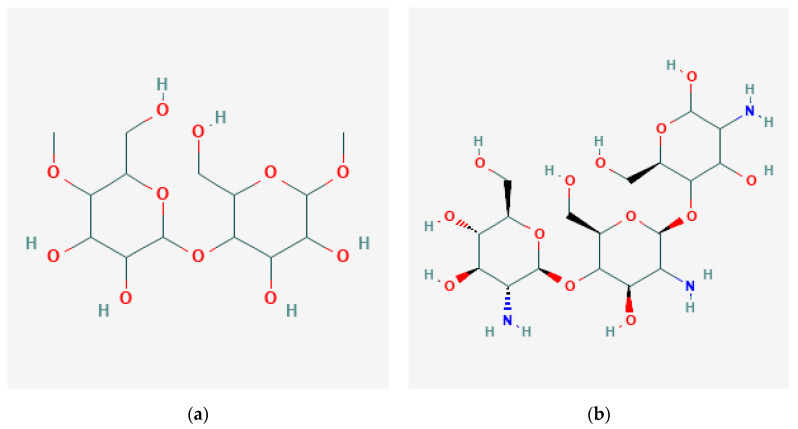

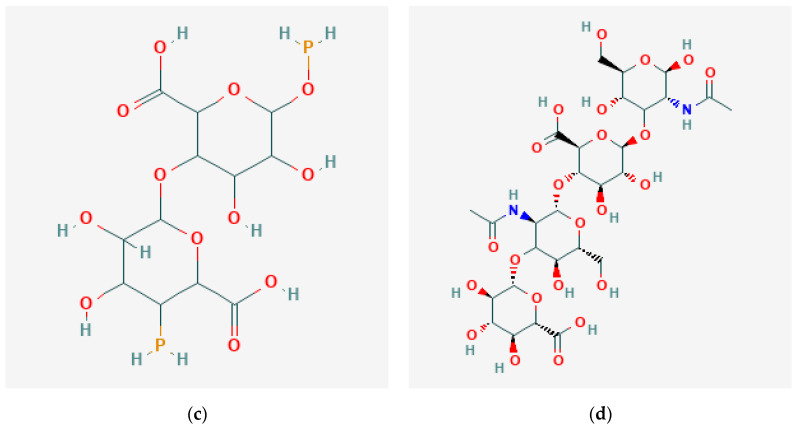
Molecular structure of polysaccharides: (**a**) cellulose-microcrystalline [120]; (**b**) chitosan [121]; (**c**) alginate [122]; (**d**) hyaluronic acid [123].

Alginate is a seaweed-derived polysaccharide extracted from Phaeophyceae-brown algae. Alginate comprises β-(1–4)-d-mannuronic acid and α-(1,4)-l-guluronic acid connected as repeated linear chains [66,117]. Alginate displays biocompatibility, biodegradability, a simple production process, and tunable mechanical properties, leaping to join in developing scaffolds in cartilage tissue engineering [118]. Moreover, alginate is hydrophilic, so it is used in wound dressing to absorb the pus and help it heal. It is also used in cell growth scaffolds, supporting blood vessels’ formation, healing bone injuries, cartilage regeneration, and drug delivery systems [119]. Alginate-based scaffolds are widely used in various tissues or organs, including skeletal muscles, pancreas, nerve, liver, and dental tissue engineering [117]. For cardiac repair, Rosellini. E. et al. produced a scaffold using alginate, elastin, and gelatin, which successfully attained the desired cellular response [103]. The molecular structure of some polysaccharides is shown in Figure 5.

#### 2.4.4. Starch

Starch is a popular polysaccharide produced by plants for energy storage. It consists of amylose and amylopectin. Amylose (a linear polymer linked by α (1–4) linkages) is connected to amylopectin (highly branched polymer) by α (1–6) linkages. Starch is highly porous and allows cells to penetrate vascularization and tissue growth. Biocompatibility, biodegradation, osteoconduction, and osteo production are some characteristics that display starch to apply in tissue engineering [124].

**Figure 6:**
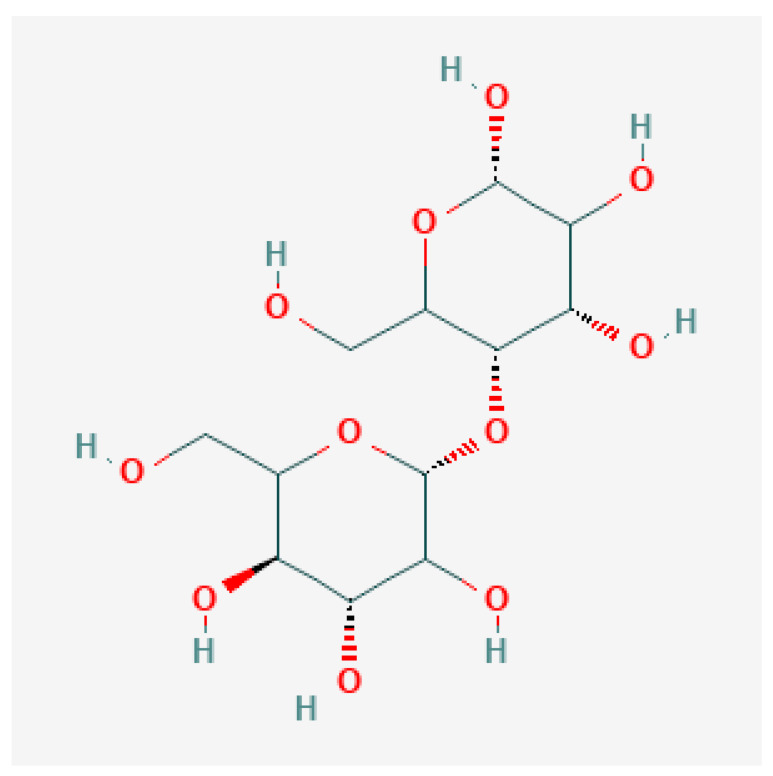
Molecular structure of starch soluble [126]. (Starch is a polysaccharide mainly found in plant cells.)

A study revealed that starch membrane, collagen, and chitosan enhance epithelial tissue regeneration during wound healing, clearly showing that starch-based scaffolds have more significance in wound healing [125]. It also helps in cell adhesion, growth, proliferation, and differentiation. Starch generally shows poor mechanical properties in aqueous media and is easily dissolved. Starch was incorporated with bio-additives to attain good mechanical properties to overcome this problem. For instance, researchers combined starch-based scaffolds with bio-additives such as citric acid, cellulose nanofibers, and hydroxyapatite to obtain the desired result. Many in vivo and in vitro assessments certified that starch-based scaffolds are better for bone regeneration [86]. The molecular structure of starch is shown in Figure 6.

#### 2.4.5. Hyaluronic Acid

Hyaluronic acid is a glycosaminoglycan that deficits sulfate bonds commonly secreted by chondrocytes and fibroblasts. It comprises repeated β-1,4-D-glucuronic acid and β-1,3-N-acetyl-D-glucosamine disaccharide units. It is mainly present in the synovial fluid, connective tissues of the dermis, the vitreum, and the dental pulp matrix. It maintains the viscoelasticity of ECM by acting as a lubricant [127]. It plays a vital role in the cell’s structural maintenance, keeps tissue hydrated, and helps cell signaling and wound repair. It is highly biocompatible, biodegradable, and can be easily modified chemically. Therefore, it is widely used as scaffolds in various forms such as sponges, cryogels, hydrogels, and injectable hydrogels [128,129,130]. A combination of collagen and hyaluronic acid scaffold material was used in cartilage regeneration, which plays a significant role in tissue repair. Mohammadi et al. prepared a scaffold by combining hyaluronic acid and collagen loaded with prednisolone to make a proper dosage form for cartilage repair [130]. According to Sieni et al., scaffolds based on hyaluronic acid show several more valuable features than collagen scaffolds in breast cancer treatment [131]. The advantages, disadvantages, and applications of each polysaccharide are mentioned in Table 3.

#### 2.4.6. Guar Gum

Guar gum is a galactomannan gum, a polysaccharide obtained from the seed of a leguminous plant, namely, guar beans, commonly known as cluster beans (*Cyamopsis teteragonolobha*). Easy accessibility, biodegradability, biocompatibility, non-toxicity, and non-immunogenicity are attractive features that tempt many researchers to develop scaffolds from guar gum [159].

#### 2.4.7. Pullulan

Pullulan is a polysaccharide made up of repeated maltotriose units connected by alpha (1–6) linkages obtained from fungi known as *Aureobasidium*. Pullulan plays a vital role in tissue engineering due to its adjustable property, biocompatibility, biodegradability, and adhesive nature. Oxidized pullulan was cross-linked with collagen, and scaffolds were produced for various biomedical applications [160,161,162].

#### 2.4.8. Collagen

Collagen is the critical protein in the connective tissues of animals, mainly in mammals. It is a protein with high biocompatibility and biodegradability. Therefore, it is applied in the medical field in various forms, such as a scaffold, drug carrier, and wound dressing [163]. The latest research shows that collagen obtained from marine organisms is used in multiple biomedical applications [164]. Collagen-based scaffolds are widely used in myocardial tissue engineering [137], cartilage tissue engineering [165], neural tissue engineering [166], musculoskeletal tissue engineering [167], and bone tissue engineering [48]. Massimino et al. developed a collagen-based scaffold obtained from bovine tendon for dermal regeneration applications [49]. Pericardial bovine and porcine tissue underwent TRICOL decellularization (detergent-based treatment), and decellularized pericardial scaffold containing collagen and elastin was considered a potential biomaterial for tissue replacement [52].

#### 2.4.9. Fibroin

Fibroin is protein silk produced by some larvae such as spiders, silkworms, mites, scorpions, and flies. The silk obtained from *Bombyx mori* (silkworm) and spiders such as *Araneus diadematus* and *Nephila clavipes* are widely used commercially [50]. Due to its excellent structural integrity and mechanical properties, silk fibroin-based biomaterial is used in musculoskeletal tissue engineering [168]. Hadisi et al. developed a silk fibroin-based scaffold composed of hardystonite loaded with gentamicin as an antibiotic agent to evaluate the in vitro and in vivo studies on bone tissue engineering applications [169]. According to Zakeri-Siavashani et al., fibroin-based scaffold containing keratin and vanillin particles acts as a potential antibacterial agent in skin tissue engineering [170].

#### 2.4.10. Keratin

**Figure 7:**
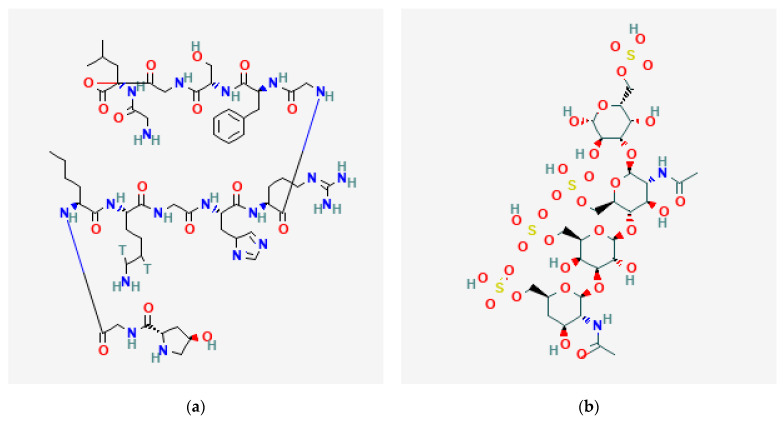

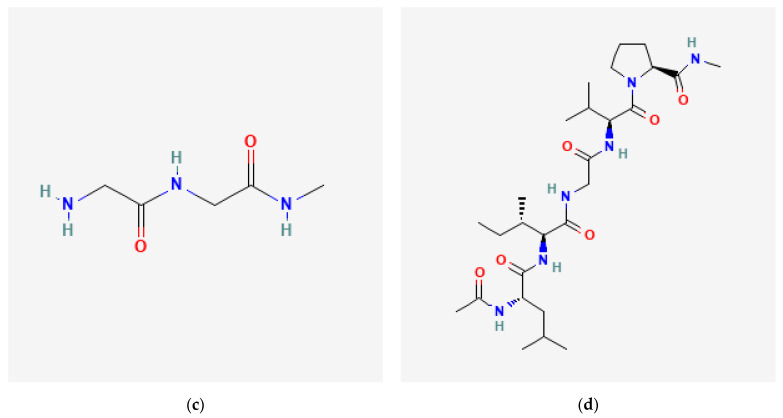
Molecular structure of some protein molecules: (**a**) collagen I [175]; (**b**) keratin [176]; (**c**) fibrin [177]; (**d**) elastin [178].

Keratin is a fibrous protein rich in cysteine and is widely present in hair, nails, wool, feathers, and horns [171]. The flexible transverse bonds in the keratin molecular chain provide suitable mechanical properties to its fibrous protein structure [172]. Keratin is insoluble, highly durable, chemically unreactive, and has binding factors that help cell adhesion and growth [173]. Keratin-based scaffolds are widely used in skin, bone, and nerve regeneration [100]. Wan et al. developed a biocomposite mat that constitutes poly (ε-caprolactone), keratin, heparin, and vascular endothelial growth factor, which acts as a well-suited scaffold in vascular tissue engineering [174]. The molecular structure of some protein molecules is shown in Figure 7.

#### 2.4.11. Elastin

Elastin is a structural protein with elastic properties widely found in connective tissue and other load-bearing tissues. Elastin is in the collagen network in many organs, including the lungs, skin, and blood vessels [179]. In vascular tissue engineering, the successful development of elastin-based vascular graft materials helps to facilitate arterial regeneration and helps to understand the macrophage-mediated immune response created after implantation [180]. Rodrigues I. C. P. et al. stated that adding elastin and collagen to his polyurethane-based scaffold improves cellular response and wettability [181]. Matriderm and glyaderm are some dermal substitutes used in wound healing made up of elastin combined with collagen, whereas matriderm constitutes bovine collagen [60].

#### 2.4.12. Fibrin

Fibrin is a protein molecule formed during blood clotting by polymerizing thrombin and fibrinogen in blood plasma. Easy fabrication, rapid biodegradability, and good biocompatibility are some properties that make fibrin used in tissue regeneration applications. It is mainly used in nerve tissue engineering, skin tissue engineering, musculoskeletal tissue engineering, and cardiac tissue engineering [182,183]. According to Bluteau et al., the low thrombin concentration increased the rate of osteoblastic marker expression. It brought out the increased angiogenic response of osteoblasts by vascular endothelial growth factor (VEGF) expression. Thus, fibrin also helps in bone tissue engineering [184].

#### 2.4.13. Gelatin

Gelatin is a protein molecule obtained by the hydrolysis of collagen, and it constitutes the Arg–Gly–Asp (RGD) peptide sequence, which helps in cell adhesion, proliferation, and differentiation [185]. The primary source of gelatin production is extracted from mammals, especially bovine hides and porcine skin [186]. Scaffold coated with gelatin inhibits complement system and opsonization. Thus, it reduces their immunogenicity [187]. In vitro studies show that scaffolds based on gelatin can control cell differentiation and gene expression [188]. Dehghan M. et al. combined gelatin, polycaprolactone, and polydimethylsiloxane to produce a scaffold, and further investigations on tests regarding biocompatibility, biodegradability, and mechanical properties gave a positive result [9].

Singh S. et al. used gelatin as a fabricating material for a cellulose-based scaffold produced from cotton to improve cell adhesion [189]. Goudarzi Z. M. et al. concluded that a poly (ε-caprolactone) and gelatin composite scaffold incorporated with acetylated cellulose nanofiber is an ideal scaffold for soft tissue engineering [190]. The list of advantages, disadvantages, and applications of each protein is mentioned in Table 4.

**Figure 8:**
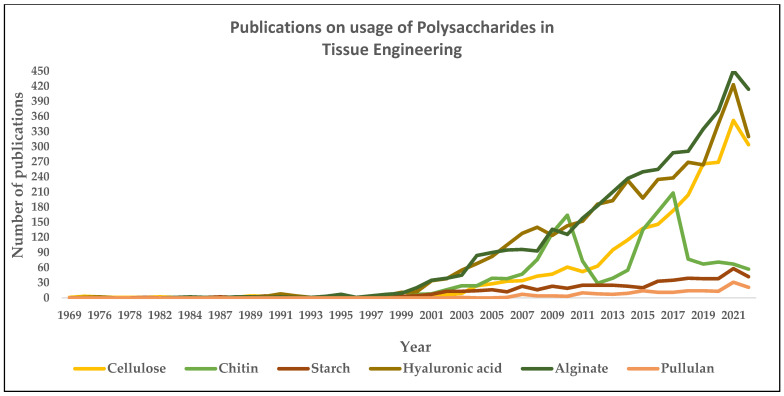
Publications on the usage of polysaccharides in tissue engineering. (The number of papers published on individual polysaccharides such as cellulose, chitin, alginate, starch, hyaluronic acid, and pullulan is drawn based on the year and the respective total number of papers published, using search engine: www.scopus.com, accessed on 15 January 2023.)

**Figure 9:**
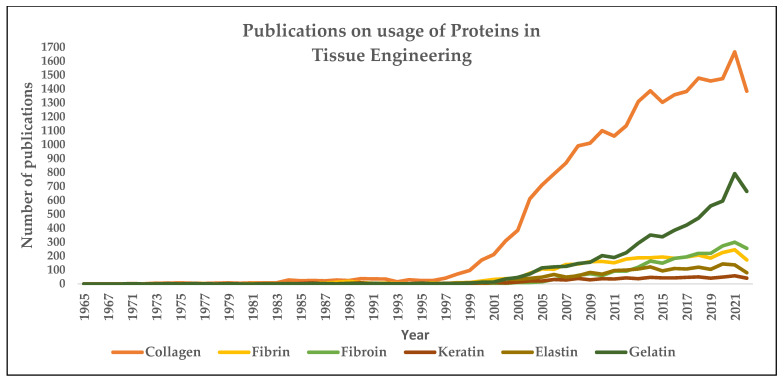
Publications on the usage of proteins in tissue engineering. (The number of papers published on individual proteins such as collagen, fibrin, fibroin, keratin, elastin, and gelatin is drawn based on the year and the respective total number of papers published, using search engine: www.scopus.com, accessed on 15 January 2023.)

Figure 8 and Figure 9 depict the number of publications on polysaccharides and proteins in tissue engineering applications. It can be seen from both figures that there is an apparent increase in terms of publications in research involving the usage of polysaccharides and proteins as a natural ingredient in developing suitable scaffolds for tissue engineering applications

### 2.5. Scaffold Fabrication Techniques

Usually, the tissue comprises repeated 3D units such as islets that act as a base for coordinating multicellular processes, maintaining mechanical properties, and integrating various organs through the circulation process. Hence, while designing the scaffold for tissue repair, we must remember that tissue substitutes should have desired mechanical properties and facilities for transporting nutrients and wastes [2]. Fabrication techniques are needed to create a proper scaffold with good mechanical properties, interconnected pores, 3D porous structure, and uniform distribution [220]. The scaffold architectural design is characterized into three levels (nano, micro, and macro) to maintain scaffold parameters such as anatomical features, cell–matrix interactions, and nutritional transportation. The nano-level architecture includes surface modification, including attachment of signaling molecules for cell adhesion, proliferation, and differentiation. Micro-level architecture constitutes pore size, porosity, interconnected pores, and spatial arrangements. The anatomical features and organ and patient specificity include macro-level architecture [221].

Fabrication techniques are classified into two categories: conventional and rapid prototyping. Techniques such as freeze drying, solvent casting, particle leaching, electro-spinning, gas foaming, and thermal-induced phase separation come under conventional fabrication techniques. These techniques are suitable for constructing porous scaffolds, but the main limitation is the lack of tunable properties to control shapes and internal architecture. In other words, achieving complex micro- and macro-level architecture is difficult in conventional fabrication techniques. Rapid prototyping is developed to overcome the drawbacks caused by conventional fabrication techniques. Rapid prototyping is known as solid free-form fabrication (SFF) and additive manufacturing (AM). It is the fastest fabrication method for assembling the desired item by using computer generation tools such as computer-aided design (CAD), magnetic resonance imaging (MRI), and computer tomography (CT). Nearly 30 rapid prototyping technologies were applied in various fields, of which 20 were used for biomedical applications [222]. Stereolithography, bioprinting, selective laser sintering, solvent-based extrusion-free forming, and fused deposition modeling are standard rapid prototyping methods used in tissue engineering for scaffold fabrication.

Usually, the primary protocol includes forming and slicing a virtual computer model ensured by layer-by-layer fabrication steps that are similar in all the various rapid prototyping techniques. Initially, a CAD model is captured or formulated from a physical unit by digital method, and then the obtained model is converted into a stereolithography file for virtual slicing. Further, it allows for digital slicing to gain cross-sectional layers. This process is termed pre-processing. Then, rapid prototyping starts to print the layer of the prototype. The post-processing steps, including surface treatment and hardening, are applied. It entirely depends on the purpose and manufacturing techniques. The desired complex micro- and macro-level architecture can also be achieved by using rapid prototyping [223].

#### 2.5.1. Freeze Drying

The freeze-drying technique is otherwise known as lyophilization or ice templating. This technique includes three steps: dissolution, solidification or freezing, and sublimation. At first, the chosen polymer is dissolved in a solvent. Secondly, the solution is loaded into a mold and placed in the freezer for solidification or freezing. It is then allowed to cool down using chemicals such as dry ice in aqueous methanol, liquid nitrogen, or mechanical refrigeration. Care should be taken at this step to maintain temperature, or else it will result in the formation of large crystals, which may affect the properties of the scaffold later. Thirdly, the sublimation process is carried out to remove water and other solvent molecules in the frozen component. This technique is highly suitable for producing scaffolds with high porosity, which provides vascularization and helps in cell proliferation and differentiation. The lyophilization method can be combined with salt leaching, gas foaming, gel casting, and liquid dispensing practices to improve the scaffold’s properties. No involvement of heat is the primary advantage of this method, so heat-sensitive molecules such as proteins or growth factors can be incorporated into it without hesitation. However, it consumes a longer time and high energy, and the cost of a freeze dryer is expensive, which are some of the drawbacks [224]. C. M. Brougham et al. developed a heart valve-shaped tissue engineering scaffold using collagen and glycosaminoglycan copolymer and fabricated it using the freeze-drying method [225]. During electro-spinning, toxic substances from organic solvents may involve scaffold preparation. Moreover, it can cause damage to the biological activity of cells. To avoid this situation, A. Izadyari Aghmiuni et al. combined freeze-drying and electro-spinning methods to develop a scaffold for tissue engineering [226].

#### 2.5.2. Solvent Casting and Particle Leaching

3D specimens with thin walls or membranes were produced using solvent casting and particle leaching methods. These thin membranes are prepared by adding salt particles to the solvent polymeric solution. Then, the solvent is allowed to evaporate, and the resulting membrane is washed with distilled water to leach out the salt. The main advantages of solvent casting and particle leaching methods are high porosity, cheapness, and straightforwardness. This technique’s usage of toxic solvents, poor interconnectivity, and irregularly shaped pores are limitations [227,228]. N. Thadavirul et al. developed a polycaprolactone porous scaffold using solvent casting and particle leaching techniques for bone tissue engineering [228]. To enhance the mechanical properties, researchers incorporate hydroxyapatite into blends of the biodegradable polymer [229].

#### 2.5.3. Gas Foaming

The gas foaming technique was introduced to avoid using organic cytotoxic solvents and high temperatures. However, the resultant material obtained had closed pores, which limited its usage, especially in cell transplantation. In this method, chosen polymer was mixed with salt particles and molded to form solid disks. Then, disks were exposed to inert gas foaming agents such as nitrogen gas or carbon dioxide with high pressure for saturation. Then, gas was decreased to ambient pressure to create thermodynamic instability, resulting in nucleation and facilitating carbon dioxide pores between polymer matrices. Finally, the salt was removed by leaching the polymer using distilled water [2,230].

#### 2.5.4. Electrospinning

**Figure 10:**
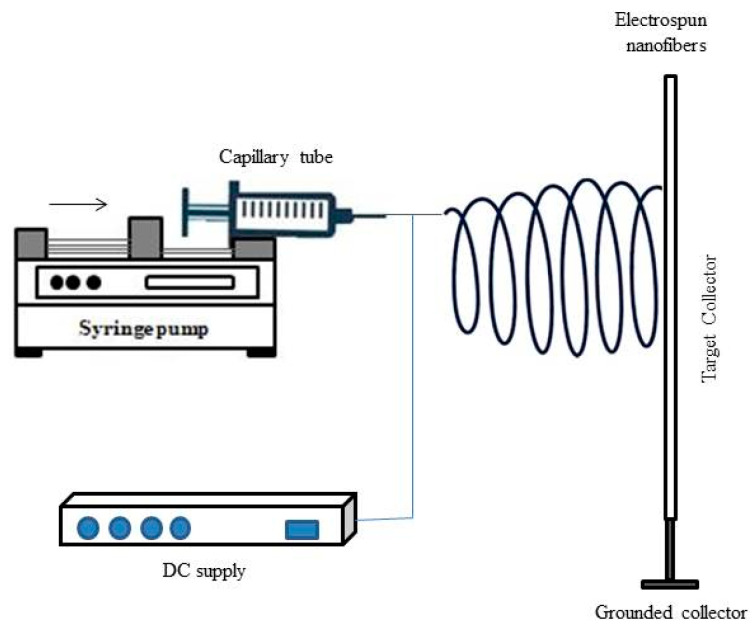
Simplified diagram of an electrospinning device, which consists of four main components such as power supply, syringe pump, metallic needle, and grounded collector. Reprinted with permission from Ref. [2]. Copyright 2019 Abdalla Eltom et al.

It is a simple technique in which solutions produce fibers by applying high-voltage electricity. The main principle behind this technique is the interaction between electrostatic repulsion and surface tension of charging liquid that receives high voltage droplets. This machine consists of four major parts: a power supply unit, a syringe pump, a metallic needle, and a grounded collector [2,231], as shown in Figure 10. Usually, this technique is widely used for producing nano-fibrous scaffolds. The liquid is injected into the capillary tube of the syringe pump. The muscle power of the electric field from a high-voltage power supply increases the surface tension of liquid extruding from the nozzle of the metallic needle.

Further, the liquid jet is continuously whipped due to electrostatic repulsion and is collected in the form of fibers in the grounded collector. Electrospinning techniques help produce scaffolds with good porosity, patterned architecture, and aligned fibers, which further help cellular response and enhance tissue regeneration. Precise control over fiber formation, homogeneous cell distribution, and lack of cellular infiltration are drawbacks of the electrospinning method [231]. Cellulose nano fiber (CNF) scaffolds developed using potato peel waste promote the adhesion and proliferation of BALB-3T3 fibroblasts cells [232].

#### 2.5.5. Thermal-Induced Phase Separation Method

This method is widely used to fabricate microcellular foams or microporous membranes. This technique de-mixes the homogenous polymer solution into polymer-rich and poor phases by applying variant temperatures. Further, lyophilization of phase-separated polymer solution helps produce microcellular structure [233]. Adjustment of pore size can be practically made possible in this method by allowing drugs and fillers. Moreover, these particles are also homogeneously distributed within the pore size. Inadequate resolution and usage of limited materials for fabrications are the main drawbacks of this method. The phase separation technique plays a vital role in fabricating a 3D nanofibrous scaffold, and it can be highly recommended to use along with another fabricating technique such as solid free form [2]. The advantages and disadvantages of various fabrication techniques are mentioned in Table 5.

#### 2.5.6. Stereolithography

**Figure 11:**
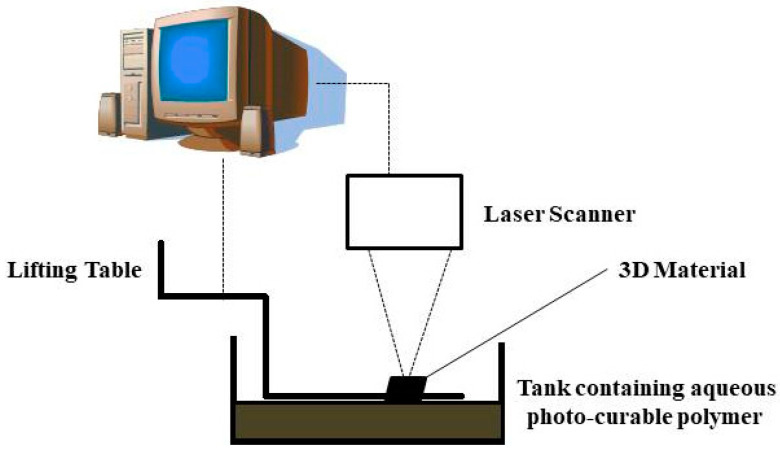
Simplified diagram of stereolithography, which consists of a tank, lifting table, laser scanner, and a computer.

Stereolithography is considered the first rapid prototype technique commercially available in the fabrication process—an aqueous photo-curable polymer was used as a raw material. An ultraviolet laser beam was used as a light source to irradiate the material surface for solidification where the untreated region remains liquid. Once the solidification of one layer is completed, the lifting table starts to move to the next layer. Subsequently, the solidified layer is recoated with new liquid resin. This photo-polymerization process is repeated until the remaining layer is done. This technique’s scaffold material has enhanced cell growth and adhesion. High resolution and uniformity in pore interconnectivity are this method’s main advantages [234]. The process involved in stereolithography is shown in Figure 11.

#### 2.5.7. Selective Laser Sintering

**Figure 12:**
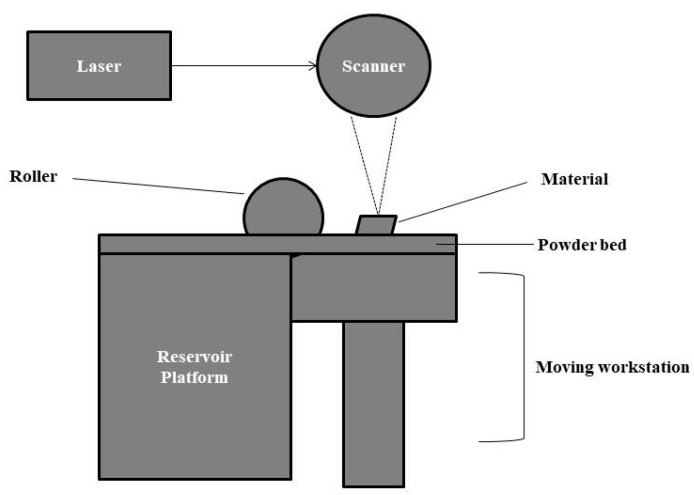
Simplified diagram of selective laser sintering, which consists of a reservoir platform, moving workstation, roller, and a scanner.

It is an additive manufacturing technique in which a high-intensity laser beam fabricates a scaffold layer-wise using computer-aided design models. Usually, materials are used in powder, and this technique can be applied to produce various materials such as ceramic, polymer, and metals. The laser beam is used to heat powder particles to glass transition temperature (near their melting point). The material was sintered to form a solid model directly without permitting the melting phase. Then, the workstation moves down layer by layer. At the same time, fresh powder is spread on the sintered object with the help of a roller, and the process is repeated until the completion of a 3D material. The scaffolds from this method provide excellent compressive strength, fracture toughness, osteoconduction, and osteoinduction. However, the high operating temperature limits the resolution, and additional procedures such as removing injected powder after processing the phase spin are some drawbacks of this method [2,234,235]. The process involved in selective laser sintering is shown in Figure 12.

#### 2.5.8. Fused Deposition Model

According to Xia et al., the fused deposition model is a filament-based additive manufacturing method. Plastic materials are used in the form of filament. They are inserted into a heating nozzle, where the filament is melted, extruded, and deposited into a plate to produce a 3D structure, layer-by-layer manner, with the help of computer-based devices. This technique is simple, cheap, versatile, and has wide applications. However, some significant deficiencies are there, too, such as difficulty in microporosity establishment, which results in a lack of cell growth and vascularization. The processing time is too long, and the heating process hinders the integration of biomolecules into the scaffold, resulting in a smooth surface unsuitable for cell adhesion, which needs further coating. Many experiments were carried out to overcome these problems, and some series were developed based on the fusion deposition model. Low-temperature deposition modeling is one of the series created, and it also gave positive responses such as better biocompatibility, biodegradability, and all required properties for bone tissue engineering [2,234,236].

#### 2.5.9. Solvent-based Extrusion 3D Printing Method

The solvent-based extrusion 3D printing method keeps biomaterials in solvents to produce inks. Then, obtained inks are extruded from the nozzle in filament to create a scaffold structure in a layer-wise manner. Natural polymers, synthetic polymers, and ceramics are the biomaterials currently being used to produce ink. This technique was widely applied to fabricate scaffolds for cartilage tissue, bone tissue, blood vessel, heart valve tissue, and skin tissue. Difficulty in obtaining appropriate levels of filament uniformity, lack of ink feasibility, and poor fidelity between the structure of computer models and printed scaffold structures are some disadvantages [237].

#### 2.5.10. Bioprinting Method

Bioprinting technology is a promising fabrication technique to develop highly mimicked tissue with digital control. A typical bioprinting method consists of pre-processing, processing, and post-processing phases. At first, in the pre-processing step, the tissue blueprint is created using computer-aided design (CAD). The vital information regarding histological structure and composition, anatomy, and human organ topology for the design can be extracted using imaging approaches. Moreover, parameters for biomaterials are also finalized during this stage. A suitable bioprinter prints the desired structure in the processing step. The bio-ink used for the bioprinter plays a crucial role in delivering the desired scaffold. Finally, post-processing steps are carried out to maturate the obtained scaffold before host implantation. Using an ideal bioreactor for the scale-up process is also under this category. Computer-aided design (CAD) and computer-aided manufacturing (CAM) are used in all three phases and play a crucial role.

Bio-CAD mimics the 3D internal structure, differentiates heterogeneous tissue types, and creates desired models. Bio-CAM is used to predict the feasibility of the fabrication process. The combination of Bio-CAD and Bio-CAM helps accelerate the bioprinting process and enhance the quality of printed tissues. The biomaterials used in this process should be printable, non-toxic, and biodegradable in vivo. Inkjet bioprinting, extrusion bioprinting, laser-assisted bioprinting, and stereolithography are the widely applied bioprinting approaches. Due to their advantages, low cost, accuracy, and high speed, bioprinting technologies have already marked their footprints in cartilage, skin, aortic valve, bone, vascular, and kidney tissues. Dependence on existing cells is the main drawback of this method [238].

#### 2.5.11. Aerosol Jet Printing

The focused airstream is used as ink instead of liquid droplets in aerosol jet printing. Either organic or inorganic materials can be used for this printing technique. A composite suspension is atomized into an aerosol using an ultrasonic or pneumatic atomizer. Then, it is transported to the deposition head by nitrogen gas, which acts as a carrier gas, and jets onto the substrate to form a 3D structure in a layer-wise manner. Polymer, ceramic, and metals can be used for aerosol jet printing. Scaffolds developed from aerosol jet printing show better cytocompatibility in in vitro studies, and it is a low-temperature process, so it is suitable for biomanufacturing too [239]. Some research on natural polymers used to fabricate scaffolds for various tissue regeneration applications is explained in Table 6.

## 3. Conclusions

Scaffolds based on natural products have gained more importance than synthetic products. The research in developing scaffolds from natural-based biomaterials for tissue regeneration applications is rapidly growing due to their outstanding properties such as promoting cell adhesion, proliferation, migration, biocompatibility, biodegradability, porosity, ease of production, inexpensive, and non-toxic. However, natural-based biomaterials have poor mechanical properties. They can be fabricated with suitable materials and used in various biomedical applications, including tissue engineering. The selection of suitable materials is crucial in tissue engineering. In that way, this paper provides a clear idea about the natural-based materials that are currently used in tissue engineering applications. In addition to that, the applications of fabrication techniques in scaffold development have been illustrated. Each technique has its respective benefits and drawbacks, and, as mentioned, appropriate selection to satisfy the need for the tissue to be repaired plays a vital role.

## Figures and Tables

**Table 1:** Young’s modulus of various tissues.

Tissue	Young’s Modulus	Reference
Bone	1–20 GPa	[26,27]
Cardiac	30–400 KPa	[27,28]
Cartilage	10–20 KPa	[27,29]
Endothelium	1–7 KPa	[27,30]
Liver	0.3–0.8 KPa	[27,31]
Lung	1–5 KPa	[27]
Nerve	0.1–2 KPa	[32]
Skin	4.6–20.0 MPa	[33]
Skeletal Muscle	20–100 KPa	[34]

**Table 2:** Recent research on the performance of the materials toward cell growth or biocompatibility.

Natural Material, Application, and References	Cells	Assay	Result
Cellulose in Bone Tissue Engineering [69]	Human osteoblast cells	MTT assay	Significant increase in cell viability of scaffold with 0.5 weight% bacterial cellulose
Cellulose in Cartilage Tissue Engineering [70]	Chondrocytes	Presto Blue^TM^ assay	Chondrocyte viability percentage in scaffold was found to be greater than 70%
Cellulose in Cardiac Tissue Engineering [71]	H9C2 rat cardiac myoblasts	MTT assay	Excellent biocompatibility in which scaffold exhibited cell proliferation and retention over the time frame of the study
Cellulose in Nerve Tissue Engineering [72]	Rat PC12 cells	Dojindo’s cell counting kit-8 (CCK-8) assay	An increase in cell viability was observed when the concentration of Poly(3-hexylthiophene)—an organic voltaic material, was up to 0.15M for the respective scaffold
Cellulose in Skin Tissue Engineering [73]	L929 mouse fibroblast	MTT assay	In vitro studies show low cell viability due to MTT assay, which is unreliable in calculating the number of cells settled inside the scaffold, but in vivo studies with Wistar rats revealed it is a promising material for diabetic wound healing
Chitosan in Bone Tissue Engineering [74]	MC3T3-E1 cells (Mouse calvaria pre-osteoblast)	Dojindo’s cell counting kit-8 (CCK-8) assay	Cell attachment, viability, and proliferation in regenerated cellulose nanofibers into chitosan hydrogel is more excellent than pure chitosan hydrogel
Chitosan in Skin Tissue Engineering [75]	Human dermal fibroblast	MTS assay	The presence of chitosan along with gelatin helps in the cellular behavior of substrates and enhances the proliferation rate of fibroblasts
Chitosan in Nerve Tissue Engineering [76]	Schwann cells	MTT assay	The rate of Schwann cell proliferation was increased after the introduction of gold nanoparticles
Chitosan in Cartilage Tissue Engineering [77]	ATDC5 (Chondrocytes)	Live/dead kit (Invitrogen)	The cells migrated toward the edges of the scaffold, and the cell population at the edges became higher. From this result, necessary modifications were carried out to develop smooth strands without any slope to encourage the cells to spread on the whole surface of scaffold
Chitosan in Cardiac Tissue Engineering [78]	H9C2 (Rat cardiac myoblast cells) and HUVEC (Human umbilical vein endothelial cells)	Alamar Blue assay	For HUVEC, more cell viability was seen in the scaffold combined with polyurethane, chitosan, and carbon nanotube than in the polyurethane scaffold and control, since polyurethane is hydrophobic and lacks enough surface of cell recognition sites, while chitosan is hydrophilic. H9C2 revealed that the developed scaffold is promising for infarcted myocardium
Chitosan in Liver Tissue Engineering [79]	HepG2 (Human hepatic carcinoma cells)	MTT assay	The P-value greater than 0.05 in all cases indicates that the scaffold is suitable for liver tissue engineering and in vivo tests
Alginate, Cellulose, and Gelatin in Bone Tissue Engineering [80]	hBMSC (Human bone marrow stromal cells)	WST-1 assay	No evidence of side effects after the cell seeding in the scaffold revealed its biocompatibility, and rapid bone regeneration was observed in the in vivo model three weeks after transplantation
Alginate in Skin Tissue Engineering [81]	Fibroblast L929 cell line	MTT assay	The resulting scaffold showed good cell adhesion based on cell concentration in the scaffold as well as the assessment of cell growth
Alginate in Cartilage Tissue Engineering [82]	AMSC (Mesenchymal stem cells derived from adipose tissue)	MTT assay	Alginate helps AMSC for chondrogenesis differentiation without any aid of exogenous differential agents
Alginate in Nerve Tissue Engineering [83]	Olfactory ecto-mesenchymal stem cells	Resazurin assay and live/dead viability assay	From the live/dead viability assay, hydrogels with 5 µm and 25 µm magnetic short fibers (MSF) and alginate ease neural-like cell proliferation. From the Resazurin assay, the cell proliferation is higher in MSF-containing hydrogel than in pure alginate
Hyaluronic Acid in Skin Tissue Engineering [84]	HDF (Human dermal fibroblast)	MTT assay	Enhanced cell proliferation was seen on the nanocomposite scaffold along with cell viability. Not only the cell proliferation but also the drug delivery was exhibited by the MTT assay
Hyaluronic Acid in Neural Tissue Engineering [85]	SH–SY5Y (Human neuroblastoma cell line)	MTT assay	The rate of SH–SY5Y proliferation is accelerated by the optimal amount of hyaluronic acid
Starch in Bone Tissue Engineering [86]	MG-63 (Human osteoblast cells)	MTT assay	Cell viability of all samples was found to be greater than 94%, which shows good cytocompatibility
Collagen in Cartilage Tissue Engineering [87]	Articular chondrocytes from new-born Sprague Dawley (SD) rats	Live/dead cell viability assay	The proportion of live cells in the collagen and sodium alginate scaffold was found to be greater than in the sodium alginate and agarose scaffold
Collagen in Corneal Tissue Engineering [88]	hBM-MSCs (Human bone marrow mesenchymal stem cells)	Cell Counting Kit-8 assay	Hydrogel combined with gelatin and collagen showed increased cell viability and proliferation with time than gelatin hydrogel
Collagen in Bone Tissue Engineering [89]	MC3T3-E1 cells from mice	Cell Counting Kit-8 assay	Collagen I proteins are relatively expressed at a higher level, promoting cell differentiation. The constructed porous microsphere had excellent biocompatibility and effectively enhanced cell adhesion, proliferation, and differentiation
Collagen in Skin Tissue Engineering [90]	ATCCR PCS-201-012 (Normal adult human dermal fibroblasts) and ATCCR PCS-200-011 (Normal primary human adult epidermal keratinocytes)	MTT assay	The scaffold made up of collagen and elastin promotes cell adhesion and proliferation
Collagen in Oral Mucosa Tissue Engineering [91]	Human primary oral fibroblast and keratinocyte cells	PrestoBlue assay	The comparative study revealed that the biological properties of the collagen-based hydrogel are superior to gelatin methacryloyl in terms of growth of oral fibroblast within the scaffold and epithelial cell differentiation and adhesion on the engineered substrate surface
Fibroin in Bone Tissue Engineering [92]	HADMSC (Human adipose-derived mesenchymal stem cells)	Live/dead assay and MTT assay	The MTT assay revealed that PRP (platelet-rich plasma)-treated composite scaffold showed a greater cell proliferation rate than untreated scaffold. The live/dead assay revealed that cells were active after day 14 on both PRP-treated and untreated scaffolds
Fibroin in Skin Tissue Engineering [93]	L929 cells	Cell Counting Kit-8 assay	In the total of 7 days, the cell proliferation rate was found to be lowest on day 3, and the cell proliferation rate increased significantly on days 5 and 7
Fibroin in Cartilage Tissue Engineering [94]	Human chondrocyte	Cell Counting Kit-8 assay and Live/dead assay	The CCK-8 assay revealed that significant cell growth was noticed from 7–14 days. From the live/dead assay, the cell viability was detected from 5–14 days
Fibroin in Corneal Tissue Engineering [95]	The limbal cells (Isolated from corneal limbus)	MTT assay	Vigorous cell adhesion and proliferation were seen on the surface of the scaffold
Fibroin in Musculoskeletal System Tissue Engineering [96]	Bone marrow-derived mesenchymal stem cells from rabbit	Live/dead assay	Silk fibroin/pullulan hydrogels contain 90% live cells after seven days of culture, which explicitly shows its good cytocompatibility
Fibroin in Neural Tissue Engineering [97]	SH-SY5Y (Human neuroblastoma cell line)	Cell Counting Kit-8 assay	Silk fibroin scaffold showed good cell survival with an increased number over time
Keratin in Bone Tissue Engineering [98]	MG-63 cells	MTT assay	Hydroxyapatite-containing scaffold showed higher cell viability
Keratin in Nerve Tissue Engineering [99]	L929 mouse lung fibroblasts, human skin fibroblasts, human Schwann cells, and human pulmonary microvascular endothelial cells	MTS assay & Alamar Blue assay	Cells seeded on keratin combined chitosan membrane showed more significant cell adhesion and metabolic activity than plain chitosan membrane
Keratin in Vascular Tissue Engineering [100]	HUVEC (Human umbilical vein endothelial cells) and HUASMC (Human umbilical arterial smooth muscle cells)	MTT assay	The developed mat had good biocompatibility, including prolonged activated partial thromboplastin time (APTT), cytocompatibility, and lower platelet adhesion. Moreover, these mats could speed up the nitric oxide generation from the donor in the blood, which accelerates endothelial cell growth, reduces smooth muscle cell proliferation, and inhibits platelet adhesion
Keratin in Skin Tissue Engineering [101]	L929 cells from mouse fibroblast	MTT assay	In vitro studies revealed cell adhesion and proliferation, whereas in vivo studies revealed wound healing
Keratin in Urethral Tissue Engineering [102]	Smooth muscle cells from rabbit	Live/dead assay	Scaffold containing calcium peroxide (CPO) displayed greater cell viability (92%–94%) than scaffold without CPO (88%–93%)
Elastin in Cardiac Tissue Engineering [103]	Cardiac progenitor cells from rats	Dil Cell Labeling	Quantitative evaluation of Dil-labelled cells occupying a fractional area after 72 h of seeding in the scaffold confirms the cell viability by in vitro studies. From the detection of immunofluorescence in the myocardium, after ten days of implant, the cell viability by in vivo study is revealed
Elastin in Vascular Tissue Engineering [104]	hAd-MSCs (Human adipose-derived mesenchymal stem cells)	MTT assay	Cell viability and proliferation were confirmed by the MTT assay, and reverse transcription-polymerase chain reaction ensures cell differentiation
Elastin in Cartilage Tissue Engineering [105]	Chondrocytes (Cartilaginous tissues of the bovine knee from calves)	XTT (2,3-bis(2-methoxy-4-nitro-5-sulfophenyl)-2H-tetrazolium-5-carboxanilide)	During implantation, the surface of the scaffold affected the cell, resulting in decreased cell activity. Then, better cell proliferation was seen after the surface modification with elastin and other materials
Gelatin in Bone Tissue Engineering [106]	MC3T3-E1 osteoblasts	Histology assay	The biocompatibility of the scaffold was determined by comparing it with Gelfoam. The cell number on the gelatin scaffold is significantly higher than on Gelfoam
Gelatin in Skin Tissue Engineering [107]	HSF (Human skin fibroblast)	MTT assay	Cells activity was not affected by the scaffold material, and it was well-suited for cell proliferation and adhesion
Gelatin in Cartilage Tissue Engineering [108]	Articular cartilage progenitor cell line	Resazurin assay	Not only the hydrophilic character of gelatin but also the presence of Arginylglycylaspartic acid (RGD)—a cell recognition domain, in its structure facilitates cell attachment
Gelatin in Nerve Tissue Engineering [109]	L929 cells from mouse fibroblast	MTT assay	Axons and neuronal dendrites formed on day 14 confirm cell differentiation along with cell viability and proliferation
Fibrin in Cartilage Tissue Engineering [110]	Human hyaline-derived chondrocytes	WST-1 assay	An increase in cellular metabolic activity with time, along with a decrease in the biomaterial volume
Fibrin in Liver Tissue Engineering [111]	HepG2 cell lines	MTT assay	From the MTT assay, the quantitative assessment of cell viability was found to be 86.75 ± 1.7%
Fibrin in Retinal Tissue Engineering [112]	ahRPE cells (Adult human retinal pigment epithelial cells)	MTT assay	Proper ahRPE cell encapsulation was done by a 84 mg/dL concentration of fibrin glue
Fibrin in Neural Tissue Engineering [113]	hEnSC (Human endometrial stem cells)	MTT assay	Novel hydrogel fabricated with fibrin, polyurethane, and multiwall carbon nanotube showed more significant cell viability and proliferation than fibrin

**Table 3:** Advantages, disadvantages, and applications of polysaccharides in various tissue regeneration applications.

Polysaccharide	Advantages	Disadvantages	Applications
Cellulose [132,133,134,135,136]	Excellent bioactivity and biocompatibility, having high mechanical properties, depends on the chosen source	Non biodegradability	Bone, tendons, cartilage, cardiovascular, muscle, neural, and skin
Chitosan [137,138,139,140,141,142,143]	Easy digestion, biocompatibility, biodegradability, antibacterial activity, and hemostatic activity	Low mechanical resistance, stiff and brittle	Bone, cartilage, skin, cardiac, muscle, liver, and nervous tissue engineering
Alginate [144,145,146,147,148]	Bioactivity, biocompatibility, biodegradability, non-immunogenicity, and non-antigenicity	Toughness, limited strength, and difficulty in controlled gelation	Bone, cartilage, Skin, and neural regeneration
Hyaluronic acid [149,150,151,152,153,154,155,156]	Bioactivity, biocompatibility, biodegradability, and easy chemical modification	Rapid degradation and poor mechanical properties	Skin and neural regeneration
Starch [124,157,158]	Biocompatibility, biodegradability, cheap, pertinent porosity	Low mechanical strength, high water uptake, difficult to process, and unstable in long-term application	Bone cement in bone defects and dental cavities

**Table 4:** Advantages, disadvantages, and applications of proteins in tissue regeneration applications.

Protein and References	Advantages	Disadvantages	Applications
Collagen [46,150,191,192,193,194,195]	Bioactive, biocompatible, biodegradable, poorly immunogenic, and mimics ECM	Poor mechanical properties	Bone, skin, dental, cornea, vascular, and cartilage regeneration
Fibroin [196,197,198,199,200,201]	Bioactivity, biocompatibility, biodegradability, low immunogenic, good mechanical properties, high tensile strength, excellent structural integrity, water-based processing, and cheap	Weak and brittle as a scaffold	Bone, skin, vascular, cartilage, tendon, hepatic, cornea, and neural regeneration, and musculoskeletal tissue engineering
Keratin [100,171,174]	Biocompatibility, biodegradability, mechanical durability	Poor mechanical properties and brittle	Skin, bone, and nerve regeneration, urinary tract and vascular tissue engineering
Elastin [202,203,204,205,206,207,208]	Bioactivity, biocompatibility, good biomechanical and biophysical properties	Difficult in sourcing, water-insoluble, difficult to manipulate in vitro, risk of contamination and inflammation	Cartilage, skin, tendon, and cardiovascular regeneration
Gelatin [209,210,211,212,213,214]	Bioactive, biocompatible, biodegradable, ECM mimicking, low immunogenic, water-soluble, and cheap	Poor mechanical properties, low solubility in concentrated aqueous media, and speed enzymatic degradation,	Bone, skin, cartilage, adipose, and neural regeneration
Fibrin [215,216,217,218,219]	Biocompatible, biodegradable, low immunogenic, and mimics ECM	Risk of contamination, expensive, poor mechanical properties, and rapid degradation rate	Cartilage, liver, retina, vascular, and neural regeneration

**Table 5:** Advantages and disadvantages of various fabrication techniques.

S.No	Techniques	Advantages	Disadvantages
1.	Freeze drying	Capability to do away with high temperatures, applicable in a variety of purposes, and pore size can be manageable to be controlled by changing the freeze-drying method	Long time consumption, high energy consumption, usage of cytotoxic solvents, and irregular pore size
2.	Solvent casting and practical leaching	Cheap and high porosity	Usage of toxic solvents
3.	Gas foaming	Absence of caustic solvents	Poor interconnectivity, low reproducibility, and structural uniformity
4.	Electrospinning	Porosity, control over morphology, and usage of simple equipment	Limited control of pore structure, use of toxic solvents, and many variables involved in the process
5.	Thermal-induced phase separation	Fast, controllable, scalable, and formation of intrinsically interconnected pores	Only used for thermoplastic
6.	Stereolithography	High resolution, fast processing, and smoother surface	Expensive, high temperature, and toxic uncured resin
7.	Selective laser sintering	Fast processing, high resolution, and no support is needed during manufacturing	High temperature, rough surface finish
8.	Fused deposition model	No requirement for solvents and good mechanical properties	Filament requirement and high temperature
9.	Solvent-based extrusion 3D printing method	Applicable to precise control of micron-level scaffold structure, suitable for ceramic and metals too	Temperature extrusion
10.	Bioprinting method	Cheap and higher accuracy	Depends on cell existence

**Table 6:** Natural polymers used to fabricate scaffolds for various tissue regeneration applications.

Natural Polymer Used and Reference	Fabrication Technique	Cell Type	Applications
Cellulose and starch [240]	Selective laser sintering	-	Drug delivery and tissue engineering
Alginate [241]	Freeze drying	MG-63 human osteosarcoma cell line	Bone tissue engineering
Chitin and chondroitin sulphate [242]	Freeze drying	Human dermal fibroblast	Skin tissue engineering
Hyaluronic acid and collagen [243]	Electrospinning	Schwann cells	Nerve regeneration
Collagen [244]	Electrospinning followed by cross-linking	H9C2 cell line from embryonic rat heart tissue	Cardiac cell therapy
Chitosan [245]	Stereolithography	Human mesenchymal stem cells	Cartilage tissue engineering
Fibrin and chitosan [246]	Electrospinning	MG-63 cell line	Bone tissue engineering
Silk fibroin [247]	3D printing	Human bronchial epithelial cell line (BEAS-2B)	Tracheal epithelial regeneration
Gelatin [248]	3D printing	MG-63 cell line	Hard tissue regeneration
Chitosan [249]	Thermal-induced phase separation	Mouse bone marrow stromal cells	Bone tissue engineering
Corn starch [250]	Solvent casting and particulate leaching	-	Bone tissue engineering
Keratin [251]	Electrospinning	Human umbilical vein endothelial cells	Vascular tissue engineering
Silk fibroin [252]	Freeze drying	Human umbilical vein endothelial cells	Skin tissue engineering
Elastin [253]	Electrospinning	MG-63 osteosarcoma cell line	Bone tissue engineering

## Data Availability

No new data were created or analyzed in this study. Data sharing is not applicable to this article.

## References

[1] Koons G.L., Diba M., Mikos A.G. (2020). Materials design for bone-tissue engineering. Nat. Rev. Mater..

[2] Eltom A., Zhong G., Muhammad A. (2019). Scaffold Techniques and Designs in Tissue Engineering Functions and Purposes: A Review. Adv. Mater. Sci. Eng..

[3] Chocholata P., Kulda V., Babuska V. (2019). Fabrication of Scaffolds for Bone-Tissue Regeneration. Materials.

[4] Lauritano D., Limongelli L., Moreo G., Favia G., Carinci F. (2020). Nanomaterials for Periodontal Tissue Engineering: Chitosan-Based Scaffolds. A Systematic Review. Nanomaterials.

[5] Tan H.-L., Kai D., Pasbakhsh P., Teow S.-Y., Lim Y.-Y., Pushpamalar J. (2020). Electrospun cellulose acetate butyrate/polyethylene glycol (CAB/PEG) composite nanofibers: A potential scaffold for tissue engineering. Colloids Surf. B Biointerfaces.

[6] Biswal T. (2020). Biopolymers for tissue engineering applications: A review. Mater. Today: Proc..

[7] Chan B.P., Leong K.W. (2008). Scaffolding in tissue engineering: General approaches and tissue-specific considerations. Eur. Spine J..

[8] Junior A.L., Pinheiro C.C.G., Fernandes T.L., Bueno D.F. (2018). The use of human dental pulp stem cells for in vivo bone tissue engineering: A systematic review. J. Tissue Eng..

[9] Dehghan M., Mehrizi M.K., Nikukar H. (2021). Modeling and optimizing a polycaprolactone/gelatin/polydimethylsiloxane nanofiber scaffold for tissue engineering: Using response surface methodology. J. Text. Inst..

[10] Dutta R.C., Dey M., Dutta A.K., Basu B. (2017). Competent processing techniques for scaffolds in tissue engineering. Biotechnol. Adv..

[11] Mathew A., Augustine R., Kalarikal N., Thomas S. (2016). Tissue Engineering: Principles, Recent Trends and the Future. Nanomedicine and Tissue Engineering.

[12] Castells-Sala C., Alemany-Ribes M., Fernández-Muiños T., Recha-Sancho L., López-Chicón P., Aloy-Reverté C., Caballero-Camino J., Márquez-Gil A., Semino C.E. (2013). Current applications of tissue engineering in biomedicine. J. Biochips Tiss. Chips.

[13] Dufey V., Tacheny A., Art M., Becken U., De Longueville F. (2016). Expansion of human bone marrow-derived mesenchymal stem cells in BioBLU 0.3 c single-use bioreactors. Appl. Note.

[14] Ye L., Swingen C., Zhang J. (2013). Induced Pluripotent Stem Cells and Their Potential for Basic and Clinical Sciences. Curr. Cardiol. Rev..

[15] Damjanov I., Damjanov I. (2009). Inflammation and Repair. Pathology Secrets.

[16] Akter F., Akter F. (2016). Principles of Tissue Engineering. Tissue Engineering Made Easy.

[17] Alaribe F.N., Manoto S.L., Motaung S.C.K.M. (2016). Scaffolds from biomaterials: Advantages and limitations in bone and tissue engineering. Biologia.

[18] Perić Kačarević Ž., Rider P., Alkildani S., Retnasingh S., Pejakić M., Schnettler R., Gosau M., Smeets R., Jung O., Barbeck M. (2019). An introduction to bone tissue engineering. Int. J. Artif. Organs.

[19] Roseti L., Parisi V., Petretta M., Cavallo C., Desando G., Bartolotti I., Grigolo B. (2017). Scaffolds for Bone Tissue Engineering: State of the art and new perspectives. Mater. Sci. Eng. C.

[20] Verma P., Verma V. (2020). Concepts of Tissue Engineering. Animal Biotechnology.

[21] Alonzo M., Primo F.A., Kumar S.A., Mudloff J.A., Dominguez E., Fregoso G., Ortiz N., Weiss W.M., Joddar B. (2020). Bone tissue engineering techniques, advances, and scaffolds for treatment of bone defects. Curr. Opin. Biomed. Eng..

[22] Roberts T.T., Rosenbaum A.J. (2012). Bone grafts, bone substitutes and orthobiologics: Bone grafts, bone substitutes and orthobiologics the bridge between basic science and clinical advancements in fracture healing. Organogenesis.

[23] Wong Y.S., Tay C.Y., Wen F., Venkatraman S.S., Tan L.P. (2012). Engineered Polymeric Biomaterials for Tissue Engineering. Curr. Tissue Eng..

[24] Nikolova M.P., Chavali M.S. (2019). Recent advances in biomaterials for 3D scaffolds: A review. Bioact. Mater..

[25] Gurumurthy B., Janorkar A.V. (2021). Improvements in mechanical properties of collagen-based scaffolds for tissue engineering. Curr. Opin. Biomed. Eng..

[26] Pal S. (2014). Mechanical Properties of Biological Materials. Design of Artificial Human Joints & Organs.

[27] Salati M.A., Khazai J., Tahmuri A.M., Samadi A., Taghizadeh A., Taghizadeh M., Zarrintaj P., Ramsey J.D., Habibzadeh S., Seidi F. (2020). Agarose-Based Biomaterials: Opportunities and Challenges in Cartilage Tissue Engineering. Polymers.

[28] Ogneva I.V., Lebedev D., Shenkman B.S. (2010). Transversal Stiffness and Young’s Modulus of Single Fibers from Rat Soleus Muscle Probed by Atomic Force Microscopy. Biophys. J..

[29] Nguyen B.V., Wang Q.G., Kuiper N.J., El Haj A.J., Thomas C.R., Zhang Z. (2010). Biomechanical properties of single chondrocytes and chondrons determined by micromanipulation and finite-element modelling. J. R. Soc. Interface.

[30] Saha K., Keung A.J., Irwin E.F., Li Y., Little L., Schaffer D.V., Healy K.E. (2008). Substrate Modulus Directs Neural Stem Cell Behavior. Biophys. J..

[31] Chen E., Novakofski J., Jenkins W., O’Brien W. (1996). Young’s modulus measurements of soft tissues with application to elasticity imaging. IEEE Trans. Ultrason. Ferroelectr. Freq. Control..

[32] Spedden E., White J.D., Naumova E.N., Kaplan D.L., Staii C. (2012). Elasticity Maps of Living Neurons Measured by Combined Fluorescence and Atomic Force Microscopy. Biophys. J..

[33] Tran T., Hamid Z., Cheong K. (2018). A Review of Mechanical Properties of Scaffold in Tissue Engineering: Aloe Vera Composites. J. Phys. Conf. Ser..

[34] Collinsworth A.M., Zhang S., Kraus W.E., Truskey G.A. (2002). Apparent elastic modulus and hysteresis of skeletal muscle cells throughout differentiation. Am. J. Physiol. Physiol..

[35] Nguyen-Truong M., Li Y., Wang Z. (2020). Mechanical Considerations of Electrospun Scaffolds for Myocardial Tissue and Regenerative Engineering. Bioengineering.

[36] Zhang X.-Y., Fang G., Zhou J. (2017). Additively Manufactured Scaffolds for Bone Tissue Engineering and the Prediction of their Mechanical Behavior: A Review. Materials.

[37] Sharma R., Kirsch R., Valente K., Perez M., Willerth S. (2021). Physical and Mechanical Characterization of Fibrin-Based Bioprinted Constructs Containing Drug-Releasing Microspheres for Neural Tissue Engineering Applications. Processes.

[38] Ezhilarasu H., Ramalingam R., Dhand C., Lakshminarayanan R., Sadiq A., Gandhimathi C., Ramakrishna S., Bay B.H., Venugopal J.R., Srinivasan D.K. (2019). Biocompatible Aloe vera and Tetracycline Hydrochloride Loaded Hybrid Nanofibrous Scaffolds for Skin Tissue Engineering. Int. J. Mol. Sci..

[39] Place E.S., Evans N.D., Stevens M.M. (2009). Complexity in biomaterials for tissue engineering. Nat. Mater..

[40] O’Brien F.J. (2011). Biomaterials & scaffolds for tissue engineering. Mater. Today.

[41] Francois E.L., Yaszemski M.J. (2019). Preclinical Bone Repair Models in Regenerative Medicine.

[42] Khan F., Tanaka M. (2018). Designing Smart Biomaterials for Tissue Engineering. Int. J. Mol. Sci..

[43] Sultana N., Hassan M.I., Lim M.M. (2015). Scaffolding Biomaterials.

[44] Asadi N., Del Bakhshayesh A.R., Davaran S., Akbarzadeh A. (2020). Common biocompatible polymeric materials for tissue engineering and regenerative medicine. Mater. Chem. Phys..

[45] Gunathilake T.M.S.U., Ching Y.C., Ching K.Y., Chuah C.H., Abdullah L.C. (2017). Biomedical and Microbiological Applications of Bio-Based Porous Materials: A Review. Polymers.

[46] Del Bakhshayesh A.R., Mostafavi E., Alizadeh E., Asadi N., Akbarzadeh A., Davaran S. (2018). Fabrication of Three-Dimensional Scaffolds Based on Nano-biomimetic Collagen Hybrid Constructs for Skin Tissue Engineering. ACS Omega.

[47] Sabir A., Abbas H., Amini A.Y., Asmal S. (2021). Characterization of duck egg shells and bioceramic materials in making denture applications. IOP Conf. Ser. Mater. Sci. Eng..

[48] Umeyama R., Yamawaki T., Liu D., Kanazawa S., Takato T., Hoshi K., Hikita A. (2020). Optimization of culture duration of bone marrow cells before transplantation with a β-tricalcium phosphate/recombinant collagen peptide hybrid scaffold. Regen. Ther..

[49] Massimino L.C., Martins V.D.C.A., Vulcani V.A.S., De Oliveira É.L., Andreeta M.B., Bonagamba T.J., Klingbeil M.F.G., Mathor M.B., de Guzzi Plepis A.M. (2020). Use of collagen and auricular cartilage in bioengineering: Scaffolds for tissue regeneration. Cell Tissue Bank..

[50] Barua E., Deoghare A.B., Deb P., Das Lala S. (2018). Naturally derived biomaterials for development of composite bone scaffold: A review. IOP Conf. Ser. Mater. Sci. Eng..

[51] Awasthi S., Pandey S.K., Arunan E., Srivastava C. (2021). A review on hydroxyapatite coatings for the biomedical applications: Experimental and theoretical perspectives. J. Mater. Chem. B.

[52] Zouhair S., Sasso E.D., Tuladhar S.R., Fidalgo C., Vedovelli L., Filippi A., Borile G., Bagno A., Marchesan M., De Rossi G. (2020). A Comprehensive Comparison of Bovine and Porcine Decellularized Pericardia: New Insights for Surgical Applications. Biomolecules.

[53] Sadat-Shojai M., Khorasani M.-T., Dinpanah-Khoshdargi E., Jamshidi A. (2013). Synthesis methods for nanosized hydroxyapatite with diverse structures. Acta Biomater..

[54] Murugiah K., Zakaria M.I., Suhaimi H., Caesarendra W., Sambudi N.S. Synthesis and Characterisation of Hydroxyapatite (HAp) from Asiatic Hard Clam (*Meretrix meretrix*) and Blood Cockle Clam (*Anadara granosa*) Using Wet Precipitation Process. Proceedings of the 2021 IEEE National Biomedical Engineering Conference (NBEC).

[55] Mohd Pu’Ad N.A.S., Koshy P., Abdullah H.Z., Idris M.I., Lee T.C. (2019). Syntheses of hydroxyapatite from natural sources. Heliyon.

[56] Karacan I., Ben-Nissan B., Sinutok S. (2019). Marine-Based Calcium Phosphates from Hard Coral and Calcified Algae for Biomedical Applications. Marine-Derived Biomaterials for Tissue Engineering Applications.

[57] Carluccio D., Demir A.G., Bermingham M.J., Dargusch M.S. (2020). Challenges and Opportunities in the Selective Laser Melting of Biodegradable Metals for Load-Bearing Bone Scaffold Applications. Met. Mater. Trans. A.

[58] Xie Y., Zhao L., Zhang Z., Wang X., Wang R., Cui C. (2018). Fabrication and properties of porous Zn-Ag alloy scaffolds as biodegradable materials. Mater. Chem. Phys..

[59] Ku S.H., Lee M., Park C.B. (2013). Carbon-Based Nanomaterials for Tissue Engineering. Adv. Healthc. Mater..

[60] Eivazzadeh-Keihan R., Maleki A., de la Guardia M., Bani M.S., Chenab K.K., Pashazadeh-Panahi P., Baradaran B., Mokhtarzadeh A., Hamblin M.R. (2019). Carbon based nanomaterials for tissue engineering of bone: Building new bone on small black scaffolds: A review. J. Adv. Res..

[61] Girotti A., Gonzalez-Valdivieso J., Santos M., Martin L., Arias F.J. (2020). Functional characterization of an enzymatically degradable multi-bioactive elastin-like recombinamer. Int. J. Biol. Macromol..

[62] Wen Q., Mithieux S.M., Weiss A.S. (2020). Elastin Biomaterials in Dermal Repair. Trends Biotechnol..

[63] Gurumurthy B., Pal P., Griggs J.A., Janorkar A.V. (2020). Optimization of collagen-elastin-like polypeptide-bioglass scaffold composition for osteogenic differentiation of adipose-derived stem cells. Materialia.

[64] Martín-Del-Campo M., Fernández-Villa D., Cabrera-Rueda G., Rojo L. (2020). Antibacterial Bio-Based Polymers for Cranio-Maxillofacial Regeneration Applications. Appl. Sci..

[65] Bedian L., Villalba-Rodríguez A.M., Hernández-Vargas G., Parra-Saldivar R., Iqbal H.M. (2017). Bio-based materials with novel characteristics for tissue engineering applications—A review. Int. J. Biol. Macromol..

[66] Diekjürgen D., Grainger D.W. (2017). Polysaccharide matrices used in 3D in vitro cell culture systems. Biomaterials.

[67] Ehrlich H., Martinović R., Joksimović D., Petrenko I., Schiaparelli S., Wysokowski M., Tsurkan D., Stelling A.L., Springer A., Gelinsky M. (2020). Conchixes: Organic scaffolds which resemble the size and shapes of mollusks shells, their isolation and potential multifunctional applications. Appl. Phys. A.

[68] Liu W., Du H., Zhang M., Liu K., Liu H., Xie H., Zhang X., Si C. (2020). Bacterial Cellulose-Based Composite Scaffolds for Biomedical Applications: A Review. ACS Sustain. Chem. Eng..

[69] Aki D., Ulag S., Unal S., Sengor M., Ekren N., Lin C.-C., Yılmazer H., Ustundag C.B., Kalaskar D.M., Gunduz O. (2020). 3D printing of PVA/hexagonal boron nitride/bacterial cellulose composite scaffolds for bone tissue engineering. Mater. Des..

[70] Namkaew J., Laowpanitchakorn P., Sawaddee N., Jirajessada S., Honsawek S., Yodmuang S. (2021). Carboxymethyl Cellulose Entrapped in a Poly(vinyl) Alcohol Network: Plant-Based Scaffolds for Cartilage Tissue Engineering. Molecules.

[71] Chen P.-H., Liao H.-C., Hsu S.-H., Chen R.-S., Wu M.-C., Yang Y.-F., Wu C.-C., Chen M.-H., Su W.-F. (2015). A novel polyurethane/cellulose fibrous scaffold for cardiac tissue engineering. RSC Adv..

[72] Zha F., Chen W., Hao L., Wu C., Lu M., Zhang L., Yu D. (2020). Electrospun cellulose-based conductive polymer nanofibrous mats: Composite scaffolds and their influence on cell behavior with electrical stimulation for nerve tissue engineering. Soft Matter.

[73] Madub K., Goonoo N., Gimié F., Arsa I.A., Schönherr H., Bhaw-Luximon A. (2021). Green seaweeds ulvan-cellulose scaffolds enhance in vitro cell growth and in vivo angiogenesis for skin tissue engineering. Carbohydr. Polym..

[74] Maharjan B., Park J., Kaliannagounder V.K., Awasthi G.P., Joshi M.K., Park C.H., Kim C.S. (2021). Regenerated cellulose nanofiber reinforced chitosan hydrogel scaffolds for bone tissue engineering. Carbohydr. Polym..

[75] Pezeshki-Modaress M., Zandi M., Rajabi S. (2018). Tailoring the gelatin/chitosan electrospun scaffold for application in skin tissue engineering: An in vitro study. Prog. Biomater..

[76] Saderi N., Rajabi M., Akbari B., Firouzi M., Hassannejad Z. (2018). Fabrication and characterization of gold nanoparticle-doped electrospun PCL/chitosan nanofibrous scaffolds for nerve tissue engineering. J. Mater. Sci. Mater. Med..

[77] Sadeghianmaryan A., Naghieh S., Sardroud H.A., Yazdanpanah Z., Soltani Y.A., Sernaglia J., Chen X. (2020). Extrusion-based printing of chitosan scaffolds and their in vitro characterization for cartilage tissue engineering. Int. J. Biol. Macromol..

[78] Ahmadi P., Nazeri N., Derakhshan M.A., Ghanbari H. (2021). Preparation and characterization of polyurethane/chitosan/CNT nanofibrous scaffold for cardiac tissue engineering. Int. J. Biol. Macromol..

[79] Ghahremanzadeh F., Alihosseini F., Semnani D. (2021). Investigation and comparison of new galactosylation methods on PCL/chitosan scaffolds for enhanced liver tissue engineering. Int. J. Biol. Macromol..

[80] Dutta S.D., Hexiu J., Patel D.K., Ganguly K., Lim K.-T. (2020). 3D-printed bioactive and biodegradable hydrogel scaffolds of alginate/gelatin/cellulose nanocrystals for tissue engineering. Int. J. Biol. Macromol..

[81] Jadbabaei S., Kolahdoozan M., Naeimi F., Ebadi-Dehaghani H. (2021). Preparation and characterization of sodium alginate–PVA polymeric scaffolds by electrospinning method for skin tissue engineering applications. RSC Adv..

[82] Shirehjini L.M., Sharifi F., Shojaei S., Irani S. (2022). Poly-caprolactone nanofibrous coated with sol-gel alginate/ mesenchymal stem cells for cartilage tissue engineering. J. Drug Deliv. Sci. Technol..

[83] Ghaderinejad P., Najmoddin N., Bagher Z., Saeed M., Karimi S., Simorgh S., Pezeshki-Modaress M. (2021). An injectable anisotropic alginate hydrogel containing oriented fibers for nerve tissue engineering. Chem. Eng. J..

[84] Ahmadi M., Mehdikhani M., Varshosaz J., Farsaei S., Torabi H. (2021). Pharmaceutical evaluation of atorvastatin-loaded nanostructured lipid carriers incorporated into the gelatin/hyaluronic acid/polycaprolactone scaffold for the skin tissue engineering. J. Biomater. Appl..

[85] Entekhabi E., Nazarpak M.H., Moztarzadeh F., Sadeghi A. (2016). Design and manufacture of neural tissue engineering scaffolds using hyaluronic acid and polycaprolactone nanofibers with controlled porosity. Mater. Sci. Eng. C.

[86] Mirab F., Eslamian M., Bagheri R. (2018). Fabrication and characterization of a starch-based nanocomposite scaffold with highly porous and gradient structure for bone tissue engineering. Biomed. Phys. Eng. Express.

[87] Yang X., Lu Z., Wu H., Li W., Zheng L., Zhao J. (2018). Collagen-alginate as bioink for three-dimensional (3D) cell printing based cartilage tissue engineering. Mater. Sci. Eng. C.

[88] Goodarzi H., Jadidi K., Pourmotabed S., Sharifi E., Aghamollaei H. (2019). Preparation and in vitro characterization of cross-linked collagen–gelatin hydrogel using EDC/NHS for corneal tissue engineering applications. Int. J. Biol. Macromol..

[89] Zhang W., Wang X.-C., Li X.-Y., Zhang L.-L., Jiang F. (2020). A 3D porous microsphere with multistage structure and component based on bacterial cellulose and collagen for bone tissue engineering. Carbohydr. Polym..

[90] Vázquez J.J., Martínez E.S.M. (2019). Collagen and elastin scaffold by electrospinning for skin tissue engineering applications. J. Mater. Res..

[91] Tabatabaei F., Moharamzadeh K., Tayebi L. (2020). Fibroblast encapsulation in gelatin methacryloyl (GelMA) versus collagen hydrogel as substrates for oral mucosa tissue engineering. J. Oral Biol. Craniofacial Res..

[92] Wei L., Wu S., Kuss M., Jiang X., Sun R., Reid P., Qin X., Duan B. (2019). 3D printing of silk fibroin-based hybrid scaffold treated with platelet rich plasma for bone tissue engineering. Bioact. Mater..

[93] Peifen M., Mengyun L., Jinglong H., Danqian L., Yan T., Liwei X., Han Z., Jianlong D., Lingyan L., Guanghui Z. (2023). New skin tissue engineering scaffold with sulfated silk fibroin/chitosan/hydroxyapatite and its application. Biochem. Biophys. Res. Commun..

[94] Hong H., Seo Y.B., Kim D.Y., Lee J.S., Lee Y.J., Lee H., Ajiteru O., Sultan T., Lee O.J., Kim S.H. (2020). Digital light processing 3D printed silk fibroin hydrogel for cartilage tissue engineering. Biomaterials.

[95] Forouzideh N., Nadri S., Fattahi A., Abdolahinia E.D., Habibizadeh M., Rostamizadeh K., Baradaran-Rafii A., Bakhshandeh H. (2020). Epigallocatechin gallate loaded electrospun silk fibroin scaffold with anti-angiogenic properties for corneal tissue engineering. J. Drug Deliv. Sci. Technol..

[96] Li T., Song X., Weng C., Wang X., Wu J., Sun L., Gong X., Zeng W.-N., Yang L., Chen C. (2018). Enzymatically crosslinked and mechanically tunable silk fibroin/pullulan hydrogels for mesenchymal stem cells delivery. Int. J. Biol. Macromol..

[97] Wang Y., Yu H., Liu H., Fan Y. (2020). Double coating of graphene oxide–polypyrrole on silk fibroin scaffolds for neural tissue engineering. J. Bioact. Compat. Polym..

[98] Sarrami P., Karbasi S., Farahbakhsh Z., Bigham A., Rafienia M. (2022). Fabrication and characterization of novel polyhydroxybutyrate-keratin/nanohydroxyapatite electrospun fibers for bone tissue engineering applications. Int. J. Biol. Macromol..

[99] Carvalho C.R., Costa J.B., Costa L., Silva-Correia J., Moay Z.K., Ng K.W., Reis R.L., Oliveira J.M. (2019). Enhanced performance of chitosan/keratin membranes with potential application in peripheral nerve repair. Biomater. Sci..

[100] Dou J., Wang Y., Jin X., Li P., Wang L., Yuan J., Shen J. (2020). PCL/sulfonated keratin mats for vascular tissue engineering scaffold with potential of catalytic nitric oxide generation. Mater. Sci. Eng. C.

[101] Ye J.-P., Gong J.-S., Su C., Liu Y.-G., Jiang M., Pan H., Li R.-Y., Geng Y., Xu Z.-H., Shi J.-S. (2020). Fabrication and characterization of high molecular keratin based nanofibrous membranes for wound healing. Colloids Surf. B Biointerfaces.

[102] Lv X., Li Z., Chen S., Xie M., Huang J., Peng X., Yang R., Wang H., Xu Y., Feng C. (2016). Structural and functional evaluation of oxygenating keratin/silk fibroin scaffold and initial assessment of their potential for urethral tissue engineering. Biomaterials.

[103] Rosellini E., Madeddu D., Barbani N., Frati C., Graiani G., Falco A., Lagrasta C., Quaini F., Cascone M.G. (2020). Development of Biomimetic Alginate/Gelatin/Elastin Sponges with Recognition Properties toward Bioactive Peptides for Cardiac Tissue Engineering. Biomimetics.

[104] Kazemi T., Mohammadpour A.A., Matin M.M., Mahdavi-Shahri N., Dehghani H., Riabi S.H.K. (2021). Decellularized bovine aorta as a promising 3D elastin scaffold for vascular tissue engineering applications. Regen. Med..

[105] Kuo Y.-C., Ku H.-F., Rajesh R. (2017). Chitosan/γ-poly(glutamic acid) scaffolds with surface-modified albumin, elastin and poly- l -lysine for cartilage tissue engineering. Mater. Sci. Eng. C.

[106] Liu X., Smith L.A., Hu J., Ma P.X. (2009). Biomimetic nanofibrous gelatin/apatite composite scaffolds for bone tissue engineering. Biomaterials.

[107] Han F., Dong Y., Su Z., Yin R., Song A., Li S. (2014). Preparation, characteristics and assessment of a novel gelatin–chitosan sponge scaffold as skin tissue engineering material. Int. J. Pharm..

[108] Semitela Â., Girão A.F., Fernandes C., Ramalho G., Bdikin I., Completo A., Marques P.A. (2020). Electrospinning of bioactive polycaprolactone-gelatin nanofibres with increased pore size for cartilage tissue engineering applications. J. Biomater. Appl..

[109] Zeinali K., Khorasani M.T., Rashidi A., Joupari M.D. (2021). Preparation and characterization of graphene oxide aerogel/gelatin as a hybrid scaffold for application in nerve tissue engineering. Int. J. Polym. Mater. Polym. Biomater..

[110] Bonhome-Espinosa A.B., Campos F., Durand-Herrera D., Sánchez-López J.D., Schaub S., Durán J.D., Lopez-Lopez M.T., Carriel V. (2020). In vitro characterization of a novel magnetic fibrin-agarose hydrogel for cartilage tissue engineering. J. Mech. Behav. Biomed. Mater..

[111] Rajalekshmi R., Shaji A.K., Joseph R., Bhatt A. (2021). Scaffold for liver tissue engineering: Exploring the potential of fibrin incorporated alginate dialdehyde–gelatin hydrogel. Int. J. Biol. Macromol..

[112] Balagholi S., Kanavi M.R., Alizadeh S., Dabbaghi R., Karami S., Kheiri B., Daftarian N. (2018). Effects of fibrin glue as a three-dimensional scaffold in cultivated adult human retinal pigment epithelial cells. J. Biomater. Appl..

[113] Hasanzadeh E., Ebrahimibarough S., Mirzaei E., Azami M., Tavangar S.M., Mahmoodi N., Basiri A., Ai J. (2019). Preparation of fibrin gel scaffolds containing MWCNT/PU nanofibers for neural tissue engineering. J. Biomed. Mater. Res. Part A.

[114] El Knidri H., Belaabed R., Addaou A., Laajeb A., Lahsini A. (2018). Extraction, chemical modification and characterization of chitin and chitosan. Int. J. Biol. Macromol..

[115] Shamshina J.L., Berton P., Rogers R.D. (2019). Advances in Functional Chitin Materials: A Review. ACS Sustain. Chem. Eng..

[116] Nezhad-Mokhtari P., Akrami-Hasan-Kohal M., Ghorbani M. (2020). An injectable chitosan-based hydrogel scaffold containing gold nanoparticles for tissue engineering applications. Int. J. Biol. Macromol..

[117] Sahoo D.R., Biswal T. (2021). Alginate and its application to tissue engineering. SN Appl. Sci..

[118] Farokhi M., Shariatzadeh F.J., Solouk A., Mirzadeh H. (2020). Alginate Based Scaffolds for Cartilage Tissue Engineering: A Review. Int. J. Polym. Mater. Polym. Biomater..

[119] Kaczmarek-Pawelska A., Pereira L. (2020). Alginate-Based Hydrogels in Regenerative Medicine. Alginate Recent Uses of This Natural Polymer.

[120] National Center for Biotechnology Information PubChem Compound Summary for CID 14055602, Cellulose, Microcrystalline. https://pubchem.ncbi.nlm.nih.gov/compound/Cellulose_-microcrystalline.

[121] National Center for Biotechnology Information PubChem Compound Summary for CID 129662530, Chitosan. https://pubchem.ncbi.nlm.nih.gov/compound/129662530.

[122] National Center for Biotechnology Information PubChem Compound Summary for CID 131704328, Alginate. https://pubchem.ncbi.nlm.nih.gov/compound/Alginate.

[123] National Center for Biotechnology Information PubChem Compound Summary for CID 24759, Hyaluronan. https://pubchem.ncbi.nlm.nih.gov/compound/Hyaluronan.

[124] Mastalska-Popławska J., Sikora M., Izak P., Góral Z. (2019). Applications of starch and its derivatives in bioceramics. J. Biomater. Appl..

[125] Waghmare V.S., Wadke P.R., Dyawanapelly S., Deshpande A., Jain R., Dandekar P. (2018). Starch based nanofibrous scaffolds for wound healing applications. Bioact. Mater..

[126] National Center for Biotechnology Information PubChem Compound Summary for CID 51003661, Starch Soluble. https://pubchem.ncbi.nlm.nih.gov/compound/Starch-soluble.

[127] Lin W., Klein J. (2021). Recent Progress in Cartilage Lubrication. Adv. Mater..

[128] Dovedytis M., Liu Z.J., Bartlett S. (2020). Hyaluronic acid and its biomedical applications: A review. Eng. Regen..

[129] Zhai P., Peng X., Li B., Liu Y., Sun H., Li X. (2020). The application of hyaluronic acid in bone regeneration. Int. J. Biol. Macromol..

[130] Mohammadi F., Samani S.M., Tanideh N., Ahmadi F. (2018). Hybrid Scaffolds of Hyaluronic Acid and Collagen Loaded with Prednisolone: An Interesting System for Osteoarthritis. Adv. Pharm. Bull..

[131] Sieni E., Dettin M., De Robertis M., Bazzolo B., Conconi M.T., Zamuner A., Marino R., Keller F., Campana L.G., Signori E. (2020). The Efficiency of Gene Electrotransfer in Breast-Cancer Cell Lines Cultured on a Novel Collagen-Free 3D Scaffold. Cancers.

[132] Zhang X., Wang C., Liao M., Dai L., Tang Y., Zhang H., Coates P., Sefat F., Zheng L., Song J. (2019). Aligned electrospun cellulose scaffolds coated with rhBMP-2 for both in vitro and in vivo bone tissue engineering. Carbohydr. Polym..

[133] Hickey R.J., Pelling A.E. (2019). Cellulose Biomaterials for Tissue Engineering. Front. Bioeng. Biotechnol..

[134] Kuzmenko V., Karabulut E., Pernevik E., Enoksson P., Gatenholm P. (2018). Tailor-made conductive inks from cellulose nanofibrils for 3D printing of neural guidelines. Carbohydr. Polym..

[135] Luo H., Cha R., Li J., Hao W., Zhang Y., Zhou F. (2019). Advances in tissue engineering of nanocellulose-based scaffolds: A review. Carbohydr. Polym..

[136] Khan S., Ul-Islam M., Ikram M., Islam S.U., Ullah M.W., Israr M., Jang J.H., Yoon S., Park J.K. (2018). Preparation and structural characterization of surface modified microporous bacterial cellulose scaffolds: A potential material for skin regeneration applications in vitro and in vivo. Int. J. Biol. Macromol..

[137] Fang Y., Zhang T., Song Y., Sun W. (2020). Assessment of various crosslinking agents on collagen/chitosan scaffolds for myocardial tissue engineering. Biomed. Mater..

[138] Maged A., Abdelkhalek A.A., Mahmoud A.A., Salah S., Ammar M.M., Ghorab M.M. (2019). Mesenchymal stem cells associated with chitosan scaffolds loaded with rosuvastatin to improve wound healing. Eur. J. Pharm. Sci..

[139] Islam M., Shahruzzaman M., Biswas S., Sakib N., Rashid T.U. (2020). Chitosan based bioactive materials in tissue engineering applications-A review. Bioact. Mater..

[140] Rodríguez-Vázquez M., Vega-Ruiz B., Ramos-Zúñiga R., Saldaña-Koppel D.A., Quiñones-Olvera L.F. (2015). Chitosan and Its Potential Use as a Scaffold for Tissue Engineering in Regenerative Medicine. BioMed Res. Int..

[141] Shabunin A.S., Yudin V.E., Dobrovolskaya I.P., Zinovyev E.V., Zubov V., Ivan’kova E.M., Morganti P. (2019). Composite Wound Dressing Based on Chitin/Chitosan Nanofibers: Processing and Biomedical Applications. Cosmetics.

[142] Sadeghi A., Moztarzadeh F., Mohandesi J.A. (2019). Investigating the effect of chitosan on hydrophilicity and bioactivity of conductive electrospun composite scaffold for neural tissue engineering. Int. J. Biol. Macromol..

[143] Niu X., Wei Y., Liu Q., Yang B., Ma N., Li Z., Zhao L., Chen W., Huang D. (2020). Silver-loaded microspheres reinforced chitosan scaffolds for skin tissue engineering. Eur. Polym. J..

[144] Naghieh S., Sarker M., Abelseth E., Chen X. (2019). Indirect 3D bioprinting and characterization of alginate scaffolds for potential nerve tissue engineering applications. J. Mech. Behav. Biomed. Mater..

[145] Deepthi S., Jayakumar R. (2018). Alginate nanobeads interspersed fibrin network as in situ forming hydrogel for soft tissue engineering. Bioact. Mater..

[146] Prasadh S., Wong R.C.W. (2018). Unraveling the mechanical strength of biomaterials used as a bone scaffold in oral and maxillofacial defects. Oral Sci. Int..

[147] Hernández-González A.C., Téllez-Jurado L., Rodríguez-Lorenzo L.M. (2019). Alginate hydrogels for bone tissue engineering, from injectables to bioprinting: A review. Carbohydr. Polym..

[148] Sun J., Tan H. (2013). Alginate-Based Biomaterials for Regenerative Medicine Applications. Materials.

[149] Huang S., Wang C., Xu J., Ma L., Gao C. (2017). In situ assembly of fibrinogen/hyaluronic acid hydrogel via knob-hole interaction for 3D cellular engineering. Bioact. Mater..

[150] Bacakova L., Pajorova J., Zikmundova M., Filova E., Mikes P., Jencova V., Kostakova E.K., Sinica A. (2019). Nanofibrous Scaffolds for Skin Tissue Engineering and Wound Healing Based on Nature-Derived Polymers. Current and Future Aspects of Nanomedicine.

[151] Monteiro I.P., Shukla A., Marques A.P., Reis R.L., Hammond P.T. (2015). Spray-assisted layer-by-layer assembly on hyaluronic acid scaffolds for skin tissue engineering. J. Biomed. Mater. Res. Part A.

[152] Chircov C., Grumezescu A.M., Bejenaru L.E. (2018). Hyaluronic acid-based scaffolds for tissue engineering. Rom. J. Morphol. Embryol..

[153] Bejoy J., Wang Z., Bijonowski B., Yang M., Ma T., Sang Q.-X., Li Y. (2018). Differential Effects of Heparin and Hyaluronic Acid on Neural Patterning of Human Induced Pluripotent Stem Cells. ACS Biomater. Sci. Eng..

[154] Movahedi M., Asefnejad A., Rafienia M., Khorasani M.T. (2020). Potential of novel electrospun core-shell structured polyurethane/starch (hyaluronic acid) nanofibers for skin tissue engineering: In vitro and in vivo evaluation. Int. J. Biol. Macromol..

[155] Spearman B.S., Agrawal N.K., Rubiano A., Simmons C.S., Mobini S., Schmidt C.E. (2020). Tunable methacrylated hyaluronic acid-based hydrogels as scaffolds for soft tissue engineering applications. J. Biomed. Mater. Res. Part A.

[156] Thompson R.E., Pardieck J., Smith L., Kenny P., Crawford L., Shoichet M., Sakiyama-Elbert S. (2018). Effect of hyaluronic acid hydrogels containing astrocyte-derived extracellular matrix and/or V2a interneurons on histologic outcomes following spinal cord injury. Biomaterials.

[157] Roslan M.R., Nasir N.F.M., Cheng E.M., Amin N.A.M. Tissue engineering scaffold based on starch: A review. Proceedings of the 2016 International Conference on Electrical, Electronics, and Optimization Techniques (ICEEOT).

[158] Espigares I., Elvira C., Mano J., Vázquez B., Román J.S., Reis R.L. (2002). New partially degradable and bioactive acrylic bone cements based on starch blends and ceramic fillers. Biomaterials.

[159] Das A., Das A., Basu A., Datta P., Gupta M., Mukherjee A. (2021). Newer guar gum ester/chicken feather keratin interact films for tissue engineering. Int. J. Biol. Macromol..

[160] Long N.S.W., Ahmad M., Hairom N.H.H., Ahmad M.B., Hairom N.H.H.B., Othman S.A.B. (2019). Tensile and Thermogravimetry Analysis of Pullulan/Cellulose Films Incorporated with Carica Papaya Seeds Extract. Materials: Technology and Applications Series 1.

[161] Selvakumar G., Lonchin S. (2020). Fabrication and characterization of collagen-oxidized pullulan scaffold for biomedical applications. Int. J. Biol. Macromol..

[162] Cavelier S. (2015). New Strategies for Bone Graft Materials. Master’s Thesis.

[163] Zarei M., Samimi A., Khorram M., Abdi M.M., Golestaneh S.I. (2021). Fabrication and characterization of conductive polypyrrole/chitosan/collagen electrospun nanofiber scaffold for tissue engineering application. Int. J. Biol. Macromol..

[164] Grabska-Zielińska S., Sionkowska A., Carvalho Â, Monteiro F. (2021). Biomaterials with Potential Use in Bone Tissue Regeneration—Collagen/Chitosan/Silk Fibroin Scaffolds Cross-Linked by EDC/NHS. Materials.

[165] Szychlinska M.A., Calabrese G., Ravalli S., Dolcimascolo A., Castrogiovanni P., Fabbi C., Puglisi C., Lauretta G., Di Rosa M., Castorina A. (2020). Evaluation of a Cell-Free Collagen Type I-Based Scaffold for Articular Cartilage Regeneration in an Orthotopic Rat Model. Materials.

[166] Jiang J.P., Liu X.Y., Zhao F., Zhu X., Li X.-Y., Niu X.G., Yao Z.T., Dai C., Xu H.-Y., Ma K. (2020). Three-dimensional bioprinting collagen/silk fibroin scaffold combined with neural stem cells promotes nerve regeneration after spinal cord injury. Neural Regen. Res..

[167] Bayrak E., Huri P.Y. (2018). Engineering Musculoskeletal Tissue Interfaces. Front. Mater..

[168] Ma D., Wang Y., Dai W. (2018). Silk fibroin-based biomaterials for musculoskeletal tissue engineering. Mater. Sci. Eng. C.

[169] Hadisi Z., Bakhsheshi-Rad H.R., Walsh T., Dehghan M.M., Farzad-Mohajeri S., Gholami H., Diyanoush A., Pagan E., Akbari M. (2020). In vitro and in vivo evaluation of silk fibroin-hardystonite-gentamicin nanofibrous scaffold for tissue engineering applications. Polym. Test..

[170] Zakeri-Siavashani A., Chamanara M., Nassireslami E., Shiri M., Hoseini-Ahmadabadi M., Paknejad B. (2020). Three dimensional spongy fibroin scaffolds containing keratin/vanillin particles as an antibacterial skin tissue engineering scaffold. Int. J. Polym. Mater. Polym. Biomater..

[171] Feroz S., Muhammad N., Ratnayake J., Dias G. (2020). Keratin-Based materials for biomedical applications. Bioact. Mater..

[172] Naderi P., Zarei M., Karbasi S., Salehi H. (2020). Evaluation of the effects of keratin on physical, mechanical and biological properties of poly (3-hydroxybutyrate) electrospun scaffold: Potential application in bone tissue engineering. Eur. Polym. J..

[173] Rojas-Martínez L., Flores-Hernandez C., López-Marín L., Martinez-Hernandez A., Thorat S., Vasquez C.R., Del Rio-Castillo A., Velasco-Santos C. (2020). 3D printing of PLA composites scaffolds reinforced with keratin and chitosan: Effect of geometry and structure. Eur. Polym. J..

[174] Wan X., Li P., Jin X., Su F., Shen J., Yuan J. (2020). Poly(ε-caprolactone)/keratin/heparin/VEGF biocomposite mats for vascular tissue engineering. J. Biomed. Mater. Res. Part A.

[175] National Center for Biotechnology Information PubChem Compound Summary for CID 6913668, Collagen I, Alpha Chain (98–110). https://pubchem.ncbi.nlm.nih.gov/compound/Collagen-I_-alpha-chain-_98-110.

[176] National Center for Biotechnology Information PubChem Compound Summary for CID 446715, Keratan. https://pubchem.ncbi.nlm.nih.gov/compound/Keratan.

[177] National Center for Biotechnology Information PubChem Compound Summary for CID 439199, Fibrin. https://pubchem.ncbi.nlm.nih.gov/compound/Fibrin.

[178] National Center for Biotechnology Information PubChem Compound Summary for CID 439221, Elastin. https://pubchem.ncbi.nlm.nih.gov/compound/Elastin.

[179] Vazquez-Portalatin N., Alfonso-Garcia A., Liu J.C., Marcu L., Panitch A. (2020). Physical, Biomechanical, and Optical Characterization of Collagen and Elastin Blend Hydrogels. Ann. Biomed. Eng..

[180] Wang Z., Liu L., Mithieux S.M., Weiss A.S. (2021). Fabricating Organized Elastin in Vascular Grafts. Trends Biotechnol..

[181] Rodrigues I.C.P., Pereira K.D., Woigt L.F., Jardini A.L., Luchessi A.D., Lopes É.S.N., Webster T.J., Gabriel L.P. (2020). A novel technique to produce tubular scaffolds based on collagen and elastin. Artif. Organs.

[182] Dubey A.P. (2021). Carbon Nanofiber and Polymer Conjugate. Carbon Nanofibers: Fundamentals and Applications.

[183] Park C.H., Woo K.M. (2018). Fibrin-Based Biomaterial Applications in Tissue Engineering and Regenerative Medicine. Adv. Exp. Med. Biol..

[184] Noori A., Ashrafi S.J., Vaez-Ghaemi R., Hatamian-Zaremi A., Webster T.J. (2017). A review of fibrin and fibrin composites for bone tissue engineering. Int. J. Nanomed..

[185] Purohit S.D., Singh H., Bhaskar R., Yadav I., Chou C.-F., Gupta M.K., Mishra N.C. (2020). Gelatin—alginate—cerium oxide nanocomposite scaffold for bone regeneration. Mater. Sci. Eng. C.

[186] Abedinia A., Nafchi A.M., Sharifi M., Ghalambor P., Oladzadabbasabadi N., Ariffin F., Huda N. (2020). Poultry gelatin: Characteristics, developments, challenges, and future outlooks as a sustainable alternative for mammalian gelatin. Trends Food Sci. Technol..

[187] Ashwin B., Abinaya B., Prasith T., Chandran S.V., Yadav L.R., Vairamani M., Patil S., Selvamurugan N. (2020). 3D-poly (lactic acid) scaffolds coated with gelatin and mucic acid for bone tissue engineering. Int. J. Biol. Macromol..

[188] Kimura A., Yoshida F., Ueno M., Taguchi M. (2021). Application of radiation crosslinking technique to development of gelatin scaffold for tissue engineering. Radiat. Phys. Chem..

[189] Singh S., Dutt D., Kaur P., Singh H., Mishra N.C. (2020). Microfibrous paper scaffold for tissue engineering application. J. Biomater. Sci. Polym. Ed..

[190] Goudarzi Z.M., Behzad T., Ghasemi-Mobarakeh L., Kharaziha M. (2021). An investigation into influence of acetylated cellulose nanofibers on properties of PCL/Gelatin electrospun nanofibrous scaffold for soft tissue engineering. Polymer.

[191] Zhang D., Wu X., Chen J., Lin K. (2018). The development of collagen based composite scaffolds for bone regeneration. Bioact. Mater..

[192] Lim Y.-S., Ok Y.-J., Hwang S.-Y., Kwak J.-Y., Yoon S. (2019). Marine Collagen as A Promising Biomaterial for Biomedical Applications. Mar. Drugs.

[193] Kim H., Jang J., Park J., Lee K.P., Lee S., Lee D.M., Kim K.H., Kim H.K., Cho D.W. (2019). Shear-induced alignment of collagen fibrils using 3D cell printing for corneal stroma tissue engineering. Biofabrication.

[194] Nabavi M.H., Salehi M., Ehterami A., Bastami F., Semyari H., Tehranchi M., Semyari H. (2020). A collagen-based hydrogel containing tacrolimus for bone tissue engineering. Drug Deliv. Transl. Res..

[195] Chen X., Hao W., Li X., Xiao Z., Yao Y., Chu Y., Farkas B., Romano I., Brandi F., Dai J. (2018). Functional Multichannel Poly(Propylene Fumarate)-Collagen Scaffold with Collagen-Binding Neurotrophic Factor 3 Promotes Neural Regeneration After Transected Spinal Cord Injury. Adv. Heal. Mater..

[196] Chen Z., Zhang Q., Li H., Wei Q., Zhao X., Chen F. (2021). Elastin-like polypeptide modified silk fibroin porous scaffold promotes osteochondral repair. Bioact. Mater..

[197] Gholipourmalekabadi M., Sapru S., Samadikuchaksaraei A., Reis R.L., Kaplan D.L., Kundu S.C. (2020). Silk fibroin for skin injury repair: Where do things stand?. Adv. Drug Deliv. Rev..

[198] Kundu B., Rajkhowa R., Kundu S.C., Wang X. (2013). Silk fibroin biomaterials for tissue regenerations. Adv. Drug Deliv. Rev..

[199] Keirouz A., Zakharova M., Kwon J., Robert C., Koutsos V., Callanan A., Chen X., Fortunato G., Radacsi N. (2020). High-throughput production of silk fibroin-based electrospun fibers as biomaterial for skin tissue engineering applications. Mater. Sci. Eng. C.

[200] Gupta P., Lorentz K.L., Haskett D.G., Cunnane E.M., Ramaswamy A.K., Weinbaum J.S., Vorp D.A., Mandal B.B. (2020). Bioresorbable silk grafts for small diameter vascular tissue engineering applications: In vitro and in vivo functional analysis. Acta Biomater..

[201] Atrian M., Kharaziha M., Emadi R., Alihosseini F. (2019). Silk-Laponite® fibrous membranes for bone tissue engineering. Appl. Clay Sci..

[202] De Torre I.G., Alonso M., Rodriguez-Cabello J.-C. (2020). Elastin-Based Materials: Promising Candidates for Cardiac Tissue Regeneration. Front. Bioeng. Biotechnol..

[203] Miranda-Nieves D., Chaikof E.L. (2017). Collagen and Elastin Biomaterials for the Fabrication of Engineered Living Tissues. ACS Biomater. Sci. Eng..

[204] Nguyen T.-U., Shojaee M., Bashur C., Kishore V. (2019). Electrochemical fabrication of a biomimetic elastin-containing bi-layered scaffold for vascular tissue engineering. Biofabrication.

[205] Wang X., Ali M.S., Lacerda C.M.R. (2018). A Three-Dimensional Collagen-Elastin Scaffold for Heart Valve Tissue Engineering. Bioengineering.

[206] Silva R., Singh R., Sarker B., Papageorgiou D.G., Juhasz-Bortuzzo J.A., Roether J.A., Cicha I., Kaschta J., Schubert D.W., Chrissafis K. (2018). Hydrogel matrices based on elastin and alginate for tissue engineering applications. Int. J. Biol. Macromol..

[207] Khalili S., Khorasani S.N., Razavi S.M., Hashemibeni B., Tamayol A. (2019). Nanofibrous Scaffolds with Biomimetic Composition for Skin Regeneration. Appl. Biochem. Biotechnol..

[208] Tian L., Prabhakaran M.P., Ramakrishna S. (2015). Strategies for regeneration of components of nervous system: Scaffolds, cells and biomolecules. Regen. Biomater..

[209] Afewerki S., Sheikhi A., Kannan S., Ahadian S., Khademhosseini A. (2019). Gelatin-polysaccharide composite scaffolds for 3D cell culture and tissue engineering: Towards natural therapeutics. Bioeng. Transl. Med..

[210] Tytgat L., Van Damme L., Van Hoorick J., Declercq H., Thienpont H., Ottevaere H., Blondeel P., Dubruel P., Van Vlierberghe S. (2019). Additive manufacturing of photo-crosslinked gelatin scaffolds for adipose tissue engineering. Acta Biomater..

[211] Ye W., Li H., Yu K., Xie C., Wang P., Zheng Y., Zhang P., Xiu J., Yang Y., He Y. (2020). 3D printing of gelatin methacrylate-based nerve guidance conduits with multiple channels. Mater. Des..

[212] Conrad B., Han L.-H., Yang F. (2018). Gelatin-Based Microribbon Hydrogels Accelerate Cartilage Formation by Mesenchymal Stem Cells in Three Dimensions. Tissue Eng. Part A.

[213] Celikkin N., Mastrogiacomo S., Jaroszewicz J., Walboomers X.F., Swieszkowski W. (2018). Gelatin methacrylate scaffold for bone tissue engineering: The influence of polymer concentration. J. Biomed. Mater. Res. Part A.

[214] Rezaeeyazdi M., Colombani T., Memic A., Bencherif S.A. (2018). Injectable Hyaluronic Acid-co-Gelatin Cryogels for Tissue-Engineering Applications. Materials.

[215] Song H.-H.G., Rumma R.T., Ozaki C.K., Edelman E.R., Chen C.S. (2018). Vascular Tissue Engineering: Progress, Challenges, and Clinical Promise. Cell Stem Cell.

[216] Abelseth E., Abelseth L., De la Vega L., Beyer S.T., Wadsworth S.J., Willerth S.M. (2019). 3D Printing of Neural Tissues Derived from Human Induced Pluripotent Stem Cells Using a Fibrin-Based Bioink. ACS Biomater. Sci. Eng..

[217] Bachmann B., Spitz S., Rothbauer M., Jordan C., Purtscher M., Zirath H., Schuller P., Eilenberger C., Ali S.F., Mühleder S. (2018). Engineering of three-dimensional pre-vascular networks within fibrin hydrogel constructs by microfluidic control over reciprocal cell signaling. Biomicrofluidics.

[218] Soleimannejad M., Ebrahimibarough S., Soleimani M., Nadri S., Tavangar S.M., Roohipoor R., Yazdankhah M., Bayat N., Riazi-Esfahani M., Ai J. (2018). Fibrin gel as a scaffold for photoreceptor cells differentiation from conjunctiva mesenchymal stem cells in retina tissue engineering. Artif. Cells Nanomed. Biotechnol..

[219] Wang X., Liu C. (2018). Fibrin Hydrogels for Endothelialized Liver Tissue Engineering with a Predesigned Vascular Network. Polymers.

[220] Zhao P., Gu H., Mi H., Rao C., Fu J., Turng L.-S. (2018). Fabrication of scaffolds in tissue engineering: A review. Front. Mech. Eng..

[221] Chia H.N., Wu B.M. (2015). Recent advances in 3D printing of biomaterials. J. Biol. Eng..

[222] Touri M., Kabirian F., Saadati M., Ramakrishna S., Mozafari M. (2019). Additive Manufacturing of Biomaterials—The Evolution of Rapid Prototyping. Adv. Eng. Mater..

[223] Yuan B., Zhou S.Y., Chen X.S. (2017). Rapid prototyping technology and its application in bone tissue engineering. J. Zhejiang Univ. Sci. B.

[224] Fereshteh Z. (2018). Freeze-drying technologies for 3D scaffold engineering. Functional 3D Tissue Engineering Scaffolds—Materials, Technologies and Applications.

[225] Brougham C.M., Levingstone T.J., Shen N., Cooney G.M., Jockenhoevel S., Flanagan T.C., O’Brien F.J. (2017). Freeze-Drying as a Novel Biofabrication Method for Achieving a Controlled Microarchitecture within Large, Complex Natural Biomaterial Scaffolds. Adv. Heal. Mater..

[226] Aghmiuni A.I., Keshel S.H., Sefat F., AkbarzadehKhiyavi A. (2021). Fabrication of 3D hybrid scaffold by combination technique of electrospinning-like and freeze-drying to create mechanotransduction signals and mimic extracellular matrix function of skin. Mater. Sci. Eng. C.

[227] Mikos A.G., Sarakinos G., Leite S.M., Vacant J.P., Langer R. (1993). Laminated three-dimensional biodegradable foams for use in tissue engineering. Biomaterials.

[228] Thadavirul N., Pavasant P., Supaphol P. (2014). Development of polycaprolactone porous scaffolds by combining solvent casting, particulate leaching, and polymer leaching techniques for bone tissue engineering. J. Biomed. Mater. Res. Part A.

[229] Prasad A., Sankar M., Katiyar V. (2017). State of Art on Solvent Casting Particulate Leaching Method for Orthopedic ScaffoldsFabrication. Mater. Today Proc..

[230] Harris L.D., Kim B., Mooney D.J. (1998). Open pore biodegradable matrices formed with gas foaming. J. Biomed. Mater. Res..

[231] Kishan A.P., Cosgriff-Hernandez E.M. (2017). Recent advancements in electrospinning design for tissue engineering applications: A review. J. Biomed. Mater. Res. Part A.

[232] Friend D.F.L., González M.E.L., Caraballo M.M., de Queiroz A.A.A. (2019). Biological properties of electrospun cellulose scaffolds from biomass. J. Biomater. Sci. Polym. Ed..

[233] Nam Y.S., Park T.G. (1999). Biodegradable polymeric microcellular foams by modified thermally induced phase separation method. Biomaterials.

[234] Melchels F.P.W., Feijen J., Grijpma D.W. (2010). A review on stereolithography and its applications in biomedical engineering. Biomaterials.

[235] Kamboj N., Ressler A., Hussainova I. (2021). Bioactive Ceramic Scaffolds for Bone Tissue Engineering by Powder Bed Selective Laser Processing: A Review. Materials.

[236] Xia X., Xu X., Lin C., Yang Y., Zeng L., Zheng Y., Wu X., Li W., Xiao L., Qian Q. (2020). Microalgal-Immobilized Biocomposite Scaffold Fabricated by Fused Deposition Modeling 3D Printing Technology for Dyes Removal. ES Mater. Manuf..

[237] Zhang B., Cristescu R., Chrisey D.B., Narayan R.J. (2019). Solvent-based Extrusion 3D Printing for the Fabrication of Tissue Engineering Scaffolds. Int. J. Bioprint..

[238] Huang Y., Zhang X.-F., Gao G., Yonezawa T., Cui X. (2017). 3D bioprinting and the current applications in tissue engineering. Biotechnol. J..

[239] Ćatić N., Wells L., Al Nahas K., Smith M., Jing Q., Keyser U.F., Cama J., Kar-Narayan S. (2020). Aerosol-jet printing facilitates the rapid prototyping of microfluidic devices with versatile geometries and precise channel functionalization. Appl. Mater. Today.

[240] Salmoria G.V., Klauss P., Paggi R.A., Kanis L.A., Lago A. (2009). Structure and mechanical properties of cellulose based scaffolds fabricated by selective laser sintering. Polym. Test..

[241] Sharmila G., Muthukumaran C., Kirthika S., Keerthana S., Kumar N.M., Jeyanthi J. (2020). Fabrication and characterization of Spinacia oleracea extract incorporated alginate/carboxymethyl cellulose microporous scaffold for bone tissue engineering. Int. J. Biol. Macromol..

[242] Deepthi S., Viha C.V.S., Thitirat C., Furuike T., Tamura H., Jayakumar R. (2014). Fabrication of Chitin/Poly(butylene succinate)/Chondroitin Sulfate Nanoparticles Ternary Composite Hydrogel Scaffold for Skin Tissue Engineering. Polymers.

[243] Entekhabi E., Nazarpak M.H., Shafieian M., Mohammadi H., Firouzi M., Hassannejad Z. (2021). Fabrication and in vitro evaluation of 3D composite scaffold based on collagen/hyaluronic acid sponge and electrospun polycaprolactone nanofibers for peripheral nerve regeneration. J. Biomed. Mater. Res. Part A.

[244] Kitsara M., Joanne P., Boitard S.E., Ben Dhiab I., Poinard B., Menasché P., Gagnieu C., Forest P., Agbulut O., Chen Y. (2015). Fabrication of cardiac patch by using electrospun collagen fibers. Microelectron. Eng..

[245] Morris V.B., Nimbalkar S., Younesi M., McClellan P., Akkus O. (2017). Mechanical Properties, Cytocompatibility and Manufacturability of Chitosan:PEGDA Hybrid-Gel Scaffolds by Stereolithography. Ann. Biomed. Eng..

[246] Rastegar A., Mahmoodi M., Mirjalili M., Nasirizadeh N. (2021). Platelet-Rich Fibrin-Loaded PCL/Chitosan Core-Shell fibers Scaffold for Enhanced Osteogenic Differentiation of Mesenchymal Stem Cells. Carbohydr. Polym..

[247] Zhong N., Dong T., Chen Z., Guo Y., Shao Z., Zhao X. (2019). A novel 3D-printed silk fibroin-based scaffold facilitates tracheal epithelium proliferation in vitro. J. Biomater. Appl..

[248] Kim H., Yang G.H., Choi C.H., Cho Y.S., Kim G. (2018). Gelatin/PVA scaffolds fabricated using a 3D-printing process employed with a low-temperature plate for hard tissue regeneration: Fabrication and characterizations. Int. J. Biol. Macromol..

[249] Chen S., Zhao X., Du C. (2018). Macroporous poly (l-lactic acid)/chitosan nanofibrous scaffolds through cloud point thermally induced phase separation for enhanced bone regeneration. Eur. Polym. J..

[250] Beh C.Y., Cheng E.M., Nasir N.F.M., Majid M.S.A., Roslan M.R.M., You K.Y., Khor S.F., Ridzuan M.J.M. (2020). Fabrication and characterization of three-dimensional porous cornstarch/n-HAp biocomposite scaffold. Bull. Mater. Sci..

[251] Li P., Wang Y., Jin X., Dou J., Han X., Wan X., Yuan J., Shen J. (2020). Fabrication of PCL/keratin composite scaffolds for vascular tissue engineering with catalytic generation of nitric oxide potential. J. Mater. Chem. B.

[252] Wang Q., Zhou S., Wang L., You R., Yan S., Zhang Q., Li M. (2021). Bioactive silk fibroin scaffold with nanoarchitecture for wound healing. Compos. Part B Eng..

[253] Hejazi F., Ebrahimi V., Asgary M., Piryaei A., Fridoni M.J., Kermani A.A., Zare F., Abdollahifar M.-A. (2021). Improved healing of critical-size femoral defect in osteoporosis rat models using 3D elastin/polycaprolactone/nHA scaffold in combination with mesenchymal stem cells. J. Mater. Sci. Mater. Med..

